# Genetically Diverse Low Pathogenicity Avian Influenza A Virus Subtypes Co-Circulate among Poultry in Bangladesh

**DOI:** 10.1371/journal.pone.0152131

**Published:** 2016-03-24

**Authors:** Nancy A. Gerloff, Salah Uddin Khan, Natosha Zanders, Amanda Balish, Najmul Haider, Ausraful Islam, Sukanta Chowdhury, Mahmudur Ziaur Rahman, Ainul Haque, Parviez Hosseini, Emily S. Gurley, Stephen P. Luby, David E. Wentworth, Ruben O. Donis, Katharine Sturm-Ramirez, C. Todd Davis

**Affiliations:** 1 Influenza Division, Centers for Disease Control and Prevention, Atlanta, GA 30333, United States of America; 2 International Centre for Diarrhoeal Disease Research, Bangladesh (icddr,b), Dhaka, Bangladesh; 3 Department of Livestock Services, Ministry of Fisheries and Livestock, Dhaka, Bangladesh; 4 EcoHealth Alliance, New York, United States of America; Linneaus University, SWEDEN

## Abstract

Influenza virus surveillance, poultry outbreak investigations and genomic sequencing were assessed to understand the ecology and evolution of low pathogenicity avian influenza (LPAI) A viruses in Bangladesh from 2007 to 2013. We analyzed 506 avian specimens collected from poultry in live bird markets and backyard flocks to identify influenza A viruses. Virus isolation-positive specimens (n = 50) were subtyped and their coding-complete genomes were sequenced. The most frequently identified subtypes among LPAI isolates were H9N2, H11N3, H4N6, and H1N1. Less frequently detected subtypes included H1N3, H2N4, H3N2, H3N6, H3N8, H4N2, H5N2, H6N1, H6N7, and H7N9. Gene sequences were compared to publicly available sequences using phylogenetic inference approaches. Among the 14 subtypes identified, the majority of viral gene segments were most closely related to poultry or wild bird viruses commonly found in Southeast Asia, Europe, and/or northern Africa. LPAI subtypes were distributed over several geographic locations in Bangladesh, and surface and internal protein gene segments clustered phylogenetically with a diverse number of viral subtypes suggesting extensive reassortment among these LPAI viruses. H9N2 subtype viruses differed from other LPAI subtypes because genes from these viruses consistently clustered together, indicating this subtype is enzootic in Bangladesh. The H9N2 strains identified in Bangladesh were phylogenetically and antigenically related to previous human-derived H9N2 viruses detected in Bangladesh representing a potential source for human infection. In contrast, the circulating LPAI H5N2 and H7N9 viruses were both phylogenetically and antigenically unrelated to H5 viruses identified previously in humans in Bangladesh and H7N9 strains isolated from humans in China. In Bangladesh, domestic poultry sold in live bird markets carried a wide range of LPAI virus subtypes and a high diversity of genotypes. These findings, combined with the seven year timeframe of sampling, indicate a continuous circulation of these viruses in the country.

## Introduction

Avian influenza virus (AIV) surveillance efforts are typically focused on viruses that represent a potential health threat to people, have a negative impact on poultry production and/or are harbored by migratory birds that could potentially carry viruses over large geographical distances. AIV are classified by 16 hemagglutinin (HA) and 9 neuraminidase (NA) surface proteins that make up a large variety of subtypes, some of which are a public health threat [1]. Multibasic amino acids stretches amino terminal to the cleavage site of the HA protein are a characteristic of highly pathogenic avian influenza (HPAI) A virus HA subtypes and these have been found in some H5 and H7 subtype viruses, which have caused significant morbidity and mortality in poultry and have occasionally infected humans [1–4]. In 2013, a low pathogenicity avian influenza (LPAI) A (H7N9) virus emerged in humans in China resulting in more than 230 deaths among approximately 680 laboratory confirmed human infections. This virus shared many genetic features common to AIV circulating in wild birds and poultry sold on live bird markets in China [5–7]. In addition, other examples of zoonotic transmission of LPAI poultry viruses include H10N8 virus in China and H6N1 in Taiwan [8–10].

Since 2007, highly pathogenic AIV (H5N1) has been enzootic in Bangladesh resulting in uninterrupted transmission in poultry along with seven confirmed human infections, one with fatal outcome [2, 4, 11–13]. Several studies highlighted the dispersal of H5N1 in the country, but there is limited information on the distribution and characteristics of LPAI viruses from that region of Asia [14–19]. In 2011, a human infection with LPAI subtype H9N2 was reported in Bangladesh [4, 11]. Although changes in the genetic and antigenic diversity and antiviral susceptibility of AIV subtypes H9N2 and H7N9 are monitored and data is regularly updated for vaccine preparedness purposes, additional information on other subtypes in poultry is needed to support risk assessment for human infection in the region [8, 20].

The territory of Bangladesh consists of a broad deltaic plain subject to frequent flooding of two major rivers, the Jamuna (Brahmaputra) and Padma (Ganges), and many tributaries. The abundance of shallow coastal waters provides a large reservoir for wildlife, especially waterfowl, which migrate from many parts of Europe and Central Asia to over-winter in the delta [21]. In addition, it is estimated that about 90% of rural households in Bangladesh raise poultry, which is an important source of food and income for the household [22–24]. Poultry includes chickens and ducks raised together and they are commonly left to scavenge for food during the day, thereby having frequent contact with wild birds [25, 26]. Hence, resident poultry are at high risk of LPAI infection and contribute to the dispersal of the vast gene pool of LPAI viruses circulating in that part of the world [27, 28]. The limited animal health resources available focus on surveillance and characterization of HPAI viruses enzootic in the country due to their substantial threat to the poultry industry and small scale poultry producers [29]. The re-occurring and unforeseen transmission events at the human-animal interface highlight the importance of continuous and active AIV surveillance in poultry paired with human influenza surveillance in densely populated regions with a high frequency of influenza virus detection, including the HPAI and LPAI co-circulation in the live bird markets [22, 29, 30].

We screened poultry specimens collected during surveillance and outbreak investigations at live bird markets and backyards in Bangladesh from 2007 through 2013. Using phylogenetic analysis of the codon-complete genomes of a subset of 50 LPAI viruses, we describe their molecular epidemiology, geographic dispersal in Bangladesh and their endemicity in poultry. In addition, we characterize the antigenicity of H9N2 and H7N9 subtype viruses by hemagglutination-inhibition assay and link these results to HA sequence analysis. When comparing these virus genomes to publicly available sequences, they demonstrate high genetic diversity and shared ancestry with viruses found primarily in wild birds.

## Results

### Sample collection, screening, and subtyping

We analyzed LPAI viruses collected in Bangladesh through seven years of surveillance and outbreak investigations starting in 2007. From the 506 pre-screened specimens received and analyzed at CDC, 367 (72.5%) were positive for influenza A virus by real-time RT-PCR (Table 1). Two-thirds of the samples (n = 222) were H5 and H9 negative, while 89 were positive for H5 and 56 for H9 subtypes. While the majority of samples were Newcastle Disease virus (NDV) negative, the highest number of NDV positive specimens (104 of 199) were found in pooled environmental swabs from Dhaka city live bird markets; a much lower number of NDV-positive samples (6 of 188) was detected in individual cloacal, oropharyngeal or tracheal swabs. Fifty viruses that were influenza A virus-positive and HPAI H5/NDV negative were successfully isolated in embryonated chicken eggs (ECEs). These isolates were further characterized by subtyping with a multiplex PCR assay. LPAI viruses belonged to 14 different subtypes; common subtypes included H9N2 (n = 12), H11N3 (n = 9), H4N6 (n = 5), and H1N1 (n = 4) (Table 2). Other subtypes, such as H1N3 (1), H2N4 (3), H3N2 (2), H3N6 (2), H3N8 (3), H4N2 (2), H7N9 (2), H5N2 (1), H6N1 (1), and H6N7 (1) were detected sporadically. Most viruses originated from poultry (domestic chickens and ducks, n = 32) or environmental specimens (n = 7) collected in live bird markets in the course of regular surveillance (Table 2). Seven AIV, subtyped H9N2, were collected during outbreak investigations (Table 2). In addition to the aforementioned viruses, we included in the codon-complete genome sequencing and phylogenetic analysis a LPAI H9N2 subtype virus isolated from a 4 year old female that tested positive for influenza A virus during community-based surveillance in Dhaka in 2011 [11].

**Table 1:** Test results for samples collected during surveillance and outbreak investigations in Bangladesh and sent to CDC for further characterization, by type of specimen and number of positives in real-time RT-PCR.

Sampling location	Collection period	Type of specimen^a^	Total	Flu A^b^	NDV^C^	Flu A/NDV	H5^d^	H9^e^
Surveillance	8/2009-2/2011	ENV P	165	135	100	86	-	36
(Live bird markets)	3/2012-10/2012	ENV P	34	17	4	8	11	1
	8/2007-4/2011	CS	106	92	4	4	13	-
	11/2012-1/2013	CS	36	27	1	-	16	-
Surveillance (Domestic ducks)	4/2011-4/2012	CS	39	16	1	8	3	-
Outbreak investigation	3/2012-1/2013	CS, TS, OP ENV P	119	74	13	5	46	18
(Poultry)	12/2009-6/2010	CS	7	6	-	-	-	1
Total (%)	8/2007-1/2013		506	367 (72.5%)	123 (24%)	111 (22%)	89 (18%)	56 (11%)

^a^CS-Cloacal swab, ENV P-Environmental pool, TS-tracheal swab, OP-oropharyngeal swab

^b^FluA-Influenza A virus

^C^NDV-Newcastle Disease virus

^d^H5-influenza A viruses with hemagglutinin H5

^e^H9-influenza A viruses with hemagglutinin H9; cut-off for positivity in all real-time RT-PCR assays was a cycle threshold (ct) of ≤37.

**Table 2:** Bangladesh low-pathogenic avian influenza viruses isolated by subtype, location within Bangladesh, collection date, accession numbers in GISAID and type of sampling.

Strain name	Subtype	Location of collection	Date of collection	Acc. no.	Type of sampling
A/duck/Bangladesh/1687/2010	H1N1	Rajshahi	16 Jul 2010	EPI540224-31	LBM
A/duck/Bangladesh/31687/2010	H1N1	Chittagong	11 Jul 2010	EPI540248-55	LBM
A/duck/Bangladesh/1592/2010	H1N1	Netrokona	20 Jan 2010	EPI540240-47	LBM
A/duck/Bangladesh/1352/2009	H1N1	Sunamgon	28 Jan 2009	EPI540232-39	LBM
A/duck/Bangladesh/1584/2010	H1N3	Netrokona	20 Jan2010	EPI540256-63	LBM
A/duck/Bangladesh/727/2011	H2N4	Sylhet	3 Apr 2011	EPI484579-80, EPI540527-32	DD
A/duck/Bangladesh/814/2011	H2N4	Sylhet	3 Apr 2011	EPI484576-77, EPI540533-38	DD
A/duck/Bangladesh/983/2011	H2N4	Sylhet	3 Apr 2011	EPI484574-75, EPI540539-44	DD
A/duck/Bangladesh/1822/2011	H3N2	Netrokona	19 Jan 2011	EPI540272-78	LBM
A/duck/Bangladesh/1025/2011	H3N2	Dinajpur	21 Feb 2011	EPI540280-87	LBM
A/duck/Bangladesh/1772/2010	H3N2	Rajshahi	12 Nov 2010	EPI540264-71	LBM
A/duck/Bangladesh/1798/2010	H3N6	Netrokona	10 Nov 2010	EPI540288-95	LBM
A/duck/Bangladesh/1800/2010	H3N6	Netrokona	10 Nov 2010	EPI540296-303	LBM
A/duck/Bangladesh/1574/2009	H3N8	Netrokona	23 Dec 2009	EPI540304-11	LBM
A/duck/Bangladesh/1575/2009	H3N8	Netrokona	23 Dec 2009	EPI540320-27	LBM
A/duck/Bangladesh/1576/2009	H3N8	Netrokona	23 Dec 2009	EPI540312-19	LBM
A/duck/Bangladesh/1745/2010	H4N2	Chittagong	17 Oct 2010	EPI540328-35	LBM
A/duck/Bangladesh/1746/2010	H4N2	Chittagong	17 Oct 2010	EPI540336-43	LBM
A/duck/Bangladesh/1766/2010	H4N6	Netrokona	27 Oct 2010	EPI540352-59	LBM
A/duck/Bangladesh/1521/2009	H4N6	Netrokona	21 Oct 2009	EPI540360-67	LBM
A/duck/Bangladesh/1283/2008	H4N6	Chittagong	21 Dec 2008	EPI540368-75	LBM
A/duck/Bangladesh/1783/2010	H4N6	Netrokona	10 Nov 2010	EPI540344-51	LBM
A/duck/Bangladesh/1784/2010	H4N6	Netrokona	10 Nov 2010	EPI540376-83	LBM
A/duck/Bangladesh/1559/2009	H5N2	Rajshahi	18 Dec 2009	EPI540384-91	LBM
A/duck/Bangladesh/1293/2008	H6N1	Rajshahi	21 Nov 2008	EPI540392-99	LBM
A/waterfowl/Bangladesh/12301/2013	H6N7	Netrokona	23 Jan 2013	EPI540400-407	LBM
A/environment/Bangladesh/1008/2010	H7N9	Dhaka	23 Sep 2010	EPI540408-15	LBM
A/environment/Bangladesh/917/2012	H7N9	Dhaka	14 Mar 2012	EPI540416-23	LBM
A/environment/Bangladesh/100/2010	H9N2	Dhaka	25 May 2010	EPI448280-87	LBM
A/environment/Bangladesh/124/2010	H9N2	Dhaka	19 Jul 2010	EPI540424-31	LBM
A/environment/Bangladesh/155/2010	H9N2	Dhaka	20 Oct 2010	EPI540432-39	LBM
A/environment/Bangladesh/177/2010	H9N2	Chittagong	12 Dec 2010	EPI540440-47	LBM
A/duck/Bangladesh/1009/2009	H9N2	Gaibanda^	5 Mar 2009	EPI457484-91	LBM
A/avian/Bangladesh/91254/2012	H9N2	Dhaka	19 Nov 2012	EPI540480-87	OI
A/avian/Bangladesh/91256/2012	H9N2	Dhaka	19 Nov 2012	EPI540464-71	OI
A/avian/Bangladesh/91277/2012	H9N2	Dhaka	20 Nov 2012	EPI540472-79	OI
A/avian/Bangladesh/91286/2012	H9N2	Dhaka	29 Nov 2012	EPI540488-95	OI
A/poultry/Bangladesh/91349/2012	H9N2	Dhaka	15 Dec 2012	EPI540456-63	OI
A/poultry/Bangladesh/91354/2012*	H9N2	Dhaka	15 Dec 2012	EPI540496-503	OI
A/poultry/Bangladesh/91311/2012	H9N2	Dhaka	15 Dec 2012	EPI540448-55	OI
A/duck/Bangladesh/1727/2010	H11N3	Natore	17 Sep 2010	EPI540168-75	LBM
A/duck/Bangladesh/1728/2010	H11N3	Natore	17 Sep 2010	EPI540176-83	LBM
A/duck/Bangladesh/1729/2010	H11N3	Natore	17 Sep 2010	EPI540184-91	LBM
A/duck/Bangladesh/1753/2010	H11N3	Netrokona	15 Sep 2010	EPI540192-99	LBM
A/duck/Bangladesh/1595/2010	H11N3	Netrokona	20 Jan 2010	EPI540152-59	LBM
A/duck/Bangladesh/1578/2009	H11N3	Netrokona	23 Dec 2009	EPI540160-67	LBM
A/duck/Bangladesh/1051/2007	H11N3	Netrokona	31 Oct 2007	EPI540208-15	LBM
A/duck/Bangladesh/1052/2007	H11N3	Netrokona	31 Oct 2007	EPI540216-23	LBM
A/environment/Bangladesh/1002/2010	H11N3	Chittagong	14 Mar 2010	EPI540200-207	LBM

*NP gene sequence not available

^Backyard poultry, DD = domestic duck grazing with wild birds, LBM = live bird market surveillance, OI = outbreak investigation

### Geographic, and source distribution

**Fig 1:**
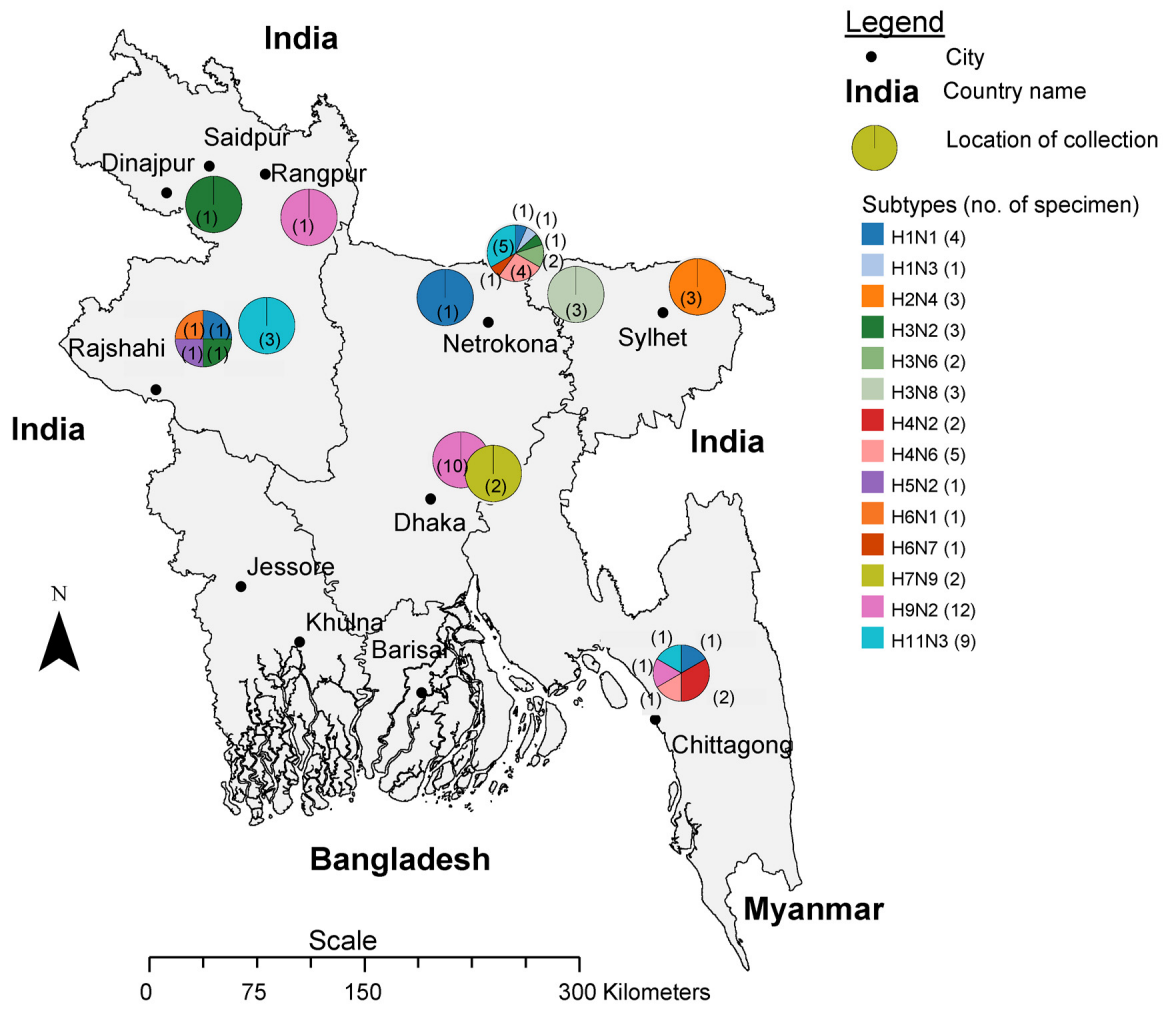
Map of Bangladesh showing the location of the collection sites. The pie charts mark the location, the subtype (color coded) and number of viruses (no. in parenthesis) that were found. The scale bar shows the distance in kilometers.

The 14 distinct subtypes of LPAI viruses were distributed throughout the sampling locations in the districts Dhaka (central Bangladesh), Chittagong (southeast), Rajshahi (west) and Dinajpur (northwest) (Fig 1). Poultry originating from all over Bangladesh is sold at these large wholesale markets (Personal communication S.U. Khan). We identified H9N2 viruses over a period of five years (2009 through 2013) from both environmental surfaces (environment) and from birds sold in Dhaka city live bird markets. Two H7N9 subtype viruses were isolated from environmental samples collected from Dhaka city live bird markets in 2010 and 2012 (Table 2). H9N2 and H11N3 subtypes were detected in both the environment or in birds at different times in bird markets in different regions in the country (Table 2, Fig 1). H2N4 subtype was found in domestic ducks that grazed in vicinity to wild birds that were sampled at Sylhet in the northeastern region (Table 2, Fig 1).

### Phylogeny of H1, H2, H3, H4, H5, H6 and H11 hemagglutinins

To investigate the genetic make-up of each virus isolate, we sequenced the coding-complete genomes of 50 LPAI viruses (Table 2). Phylogenies and nucleotide differences were analyzed individually for hemagglutinin gene segments subtype H1 through H11. Most HA gene segments clustered in groups within the same subtype showing common ancestry with viruses of South East Asian origin. Genetic analysis showed that two pairs of viruses were identical in their internal and surface protein coding gene segments (A/duck/Bangladesh/1822/2011, A/duck/Bangladesh /1025/2011, both subtype H3N2; A/duck/Bangladesh/1783/2010, A/duck/Bangladesh/1784/2010, both subtype H4N6). These samples were collected simultaneously at identical sites (Table 2).

**Fig 2:**
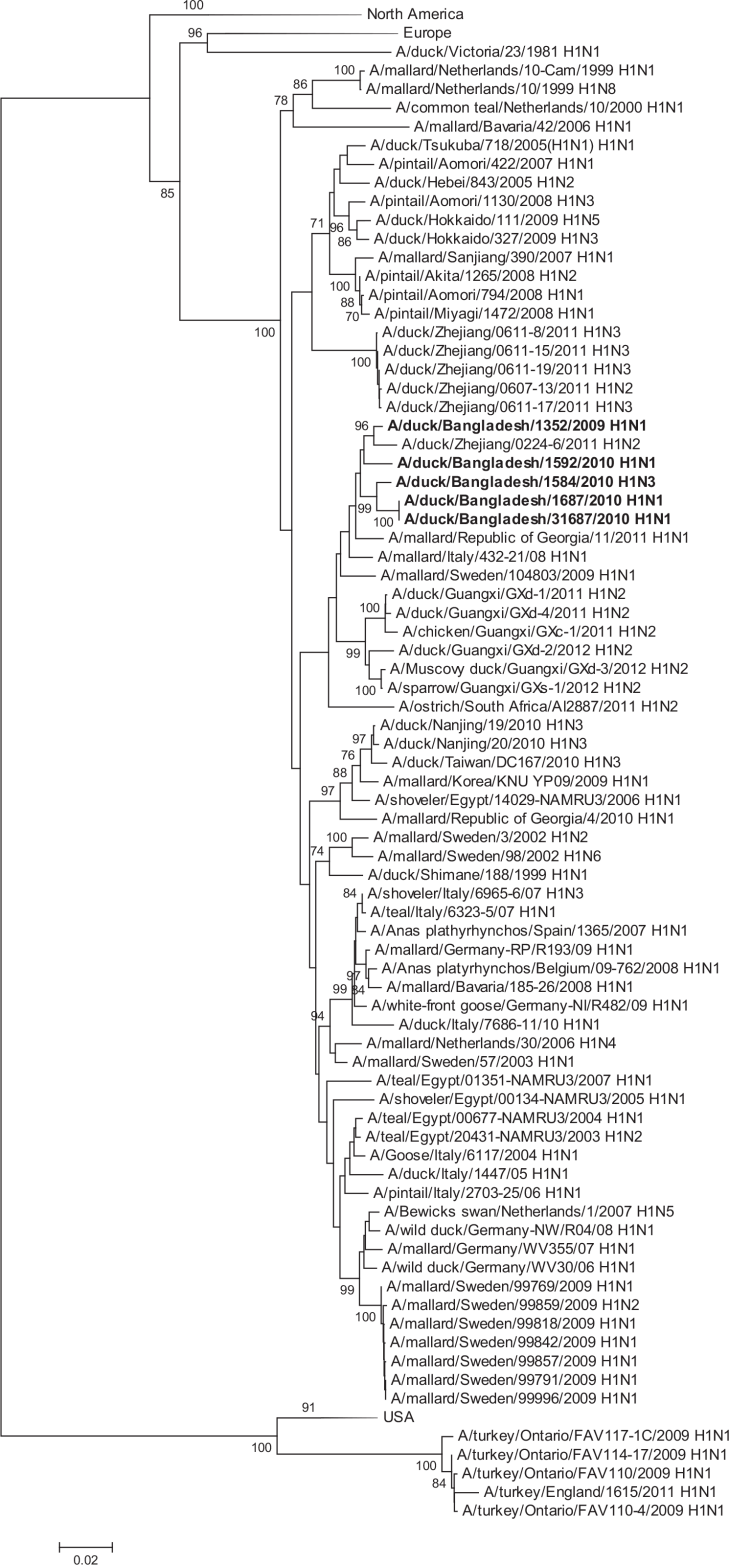
Phylogenies of the complete coding hemagglutinin genes for subtype H1. The viruses identified in this study are shown in boldface. For clarity large branches were collapsed and labeled according to the geographic location or collection years of viruses in that branch. Bootstrap values ≥70 are shown on branches.

**Fig 3:**
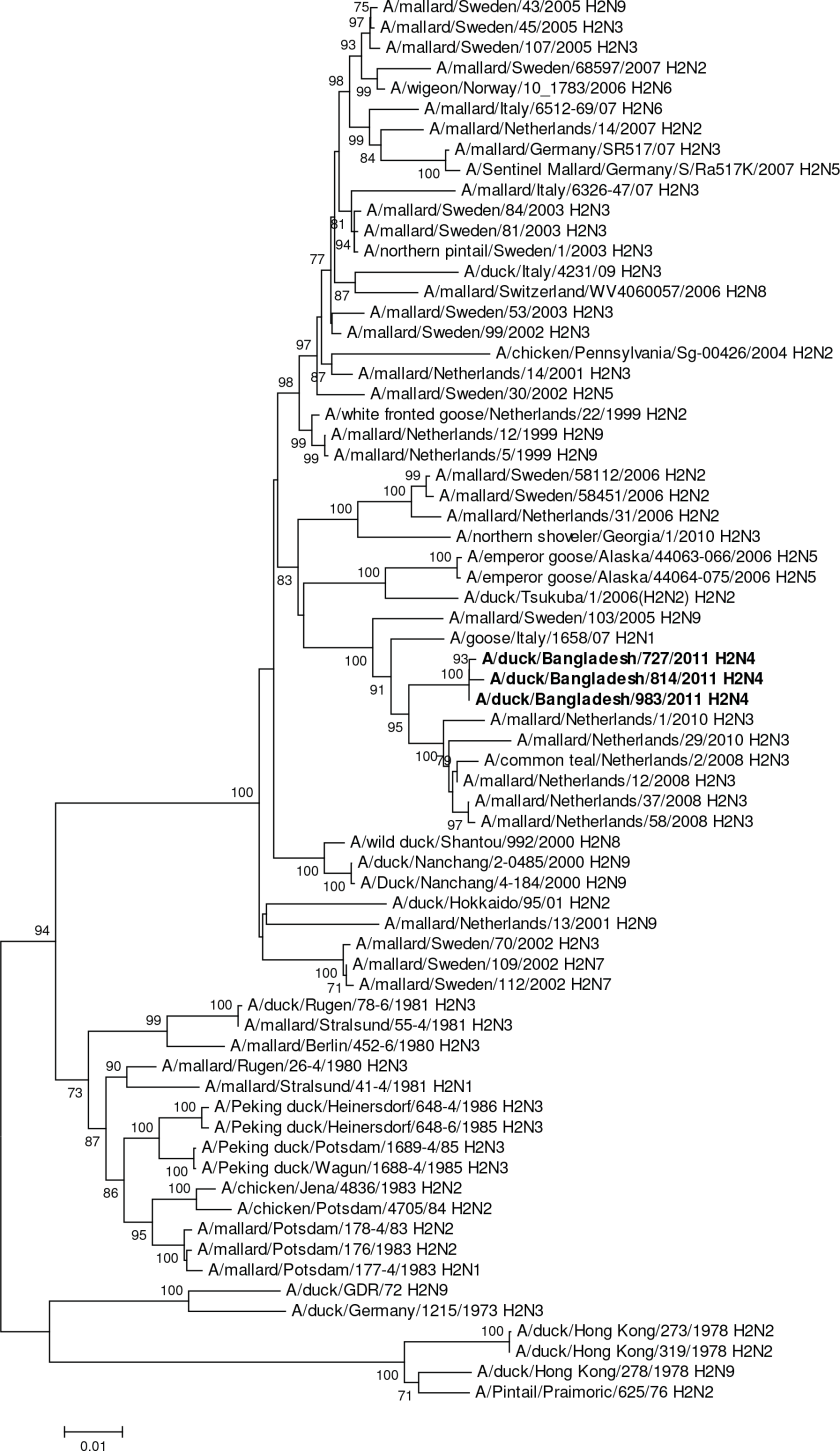
Phylogenies of the complete coding hemagglutinin genes for subtype H2. The viruses identified in this study are shown in boldface. For clarity large branches were collapsed and labeled according to the geographic location or collection years of viruses in that branch. Bootstrap values ≥70 are shown on branches.

**Fig 4:**
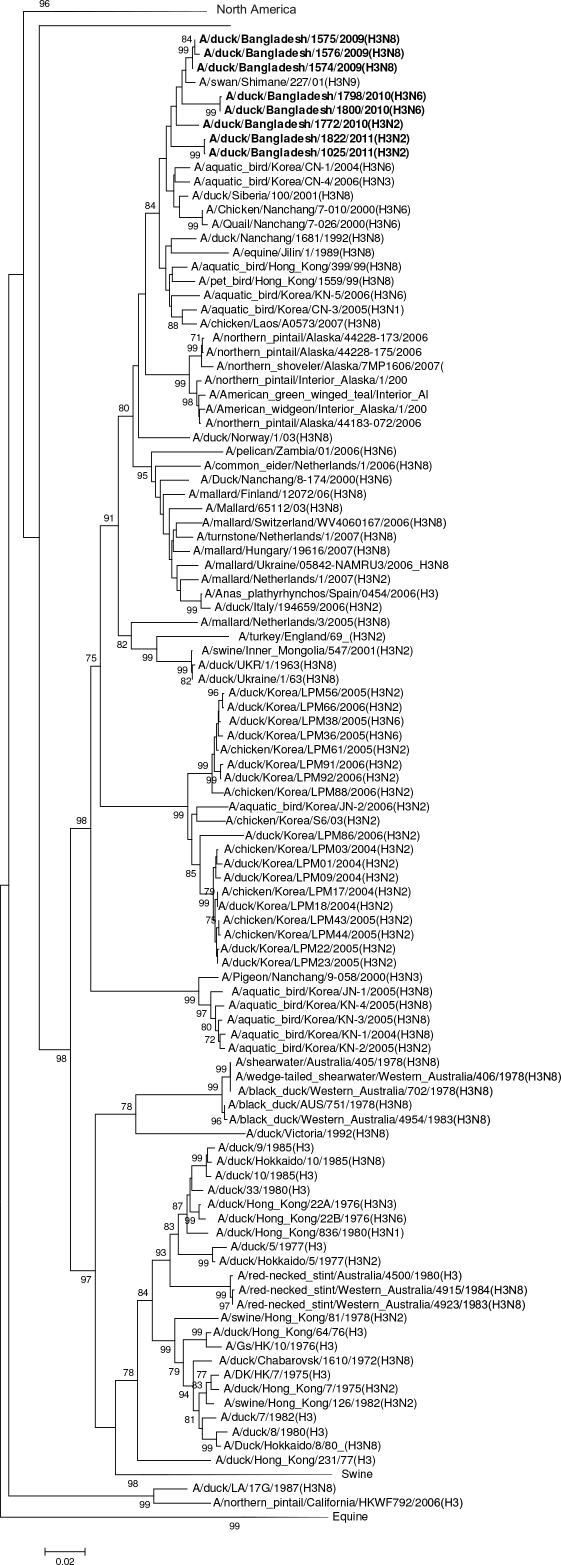
Phylogenies of the complete coding hemagglutinin genes for subtype H3. The viruses identified in this study are shown in boldface. For clarity large branches were collapsed and labeled according to the geographic location or collection years of viruses in that branch. Bootstrap values ≥70 are shown on branches.

**Fig 5:**
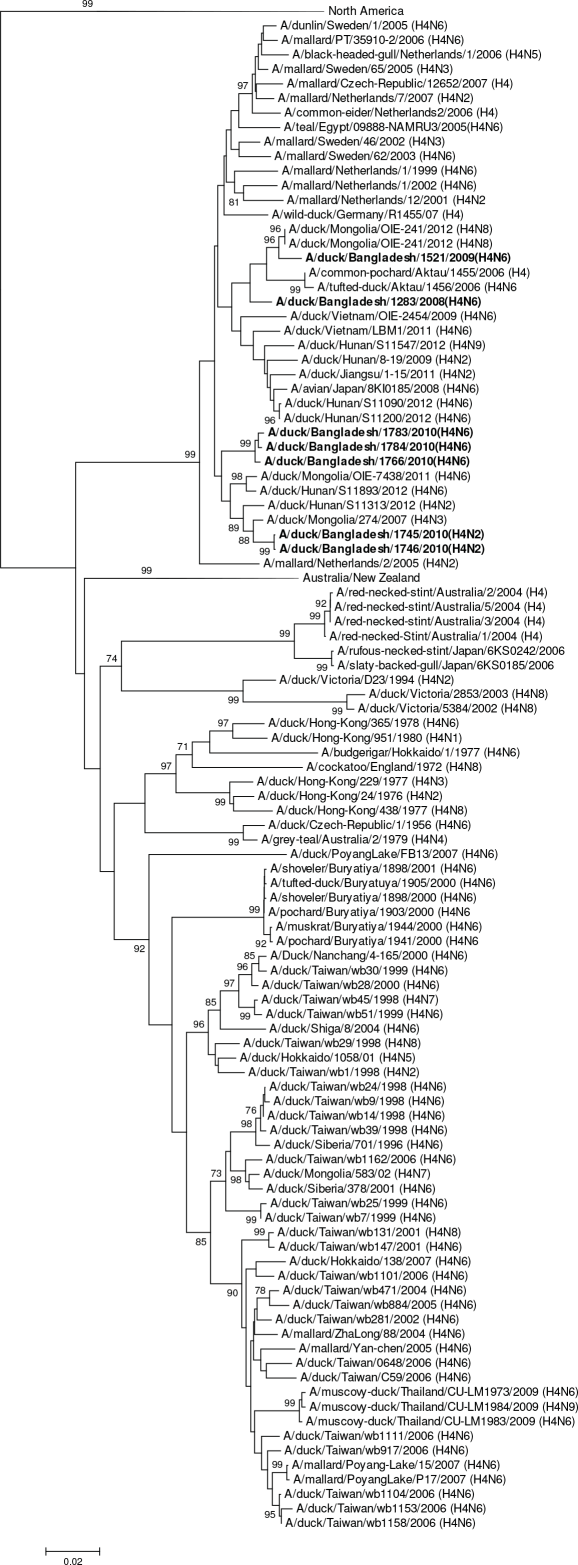
Phylogenies of the complete coding hemagglutinin genes for subtype H4. The viruses identified in this study are shown in boldface. For clarity large branches were collapsed and labeled according to the geographic location or collection years of viruses in that branch. Bootstrap values ≥70 are shown on branches.

**Fig 6:**
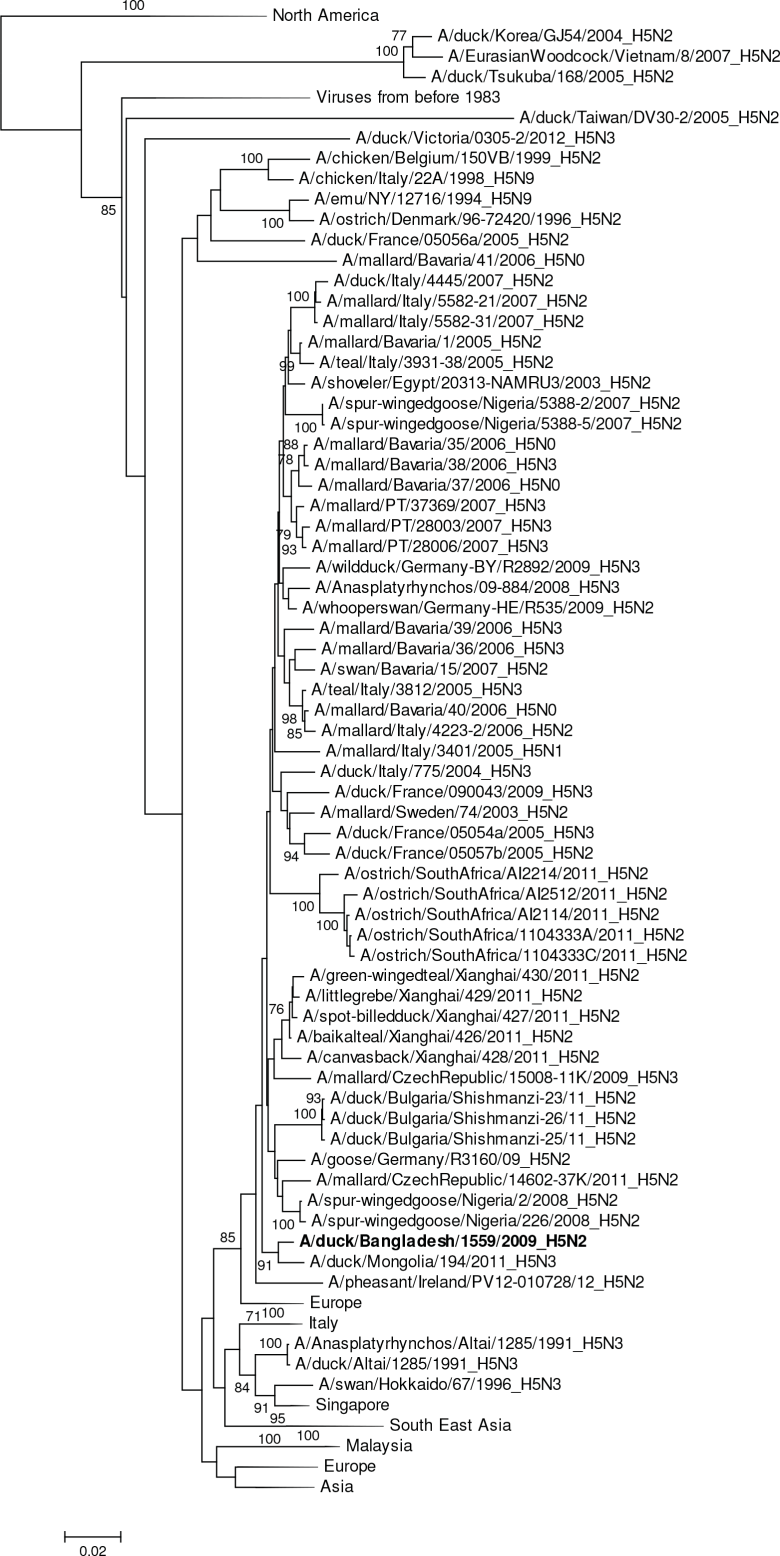
Phylogenies of the complete coding hemagglutinin genes for subtype H5. The viruses identified in this study are shown in boldface. For clarity large branches were collapsed and labeled according to the geographic location or collection years of viruses in that branch. Bootstrap values ≥70 are shown on branches.

HA gene segments of subtype H1 viruses originated from avian species and grouped in larger clusters with Asian viruses (A/duck/Zhejiang/0224-6/2011 [H1N2]), European viruses, and South African viruses (Fig 2).The HAs of H2 viruses from domestic ducks formed a distinct genetic group with viruses from Europe (Netherlands, Sweden and Italy, Fig 3). All of the H3N6 and H3N8 viruses were phylogenetically related and clustered in a group with a South East Asian virus (e.g. A/swan/Shimane/227/01 [H3N9]) and other viruses from Korea, China and Siberia (Fig 4). The HA of all H4N2 and H4N6 viruses shared a group with H4 viruses from Central, East Asia, Europe and Egypt (Fig 5). The HA of a single H5N2 virus was genetically related to central Asian viruses (e.g. A/duck/Mongolia/194/2011 [H5N3]) with high support on a shared node. However, the larger group of genetically similar viruses includes Chinese, African and European strains (Fig 6).

**Fig 7:**
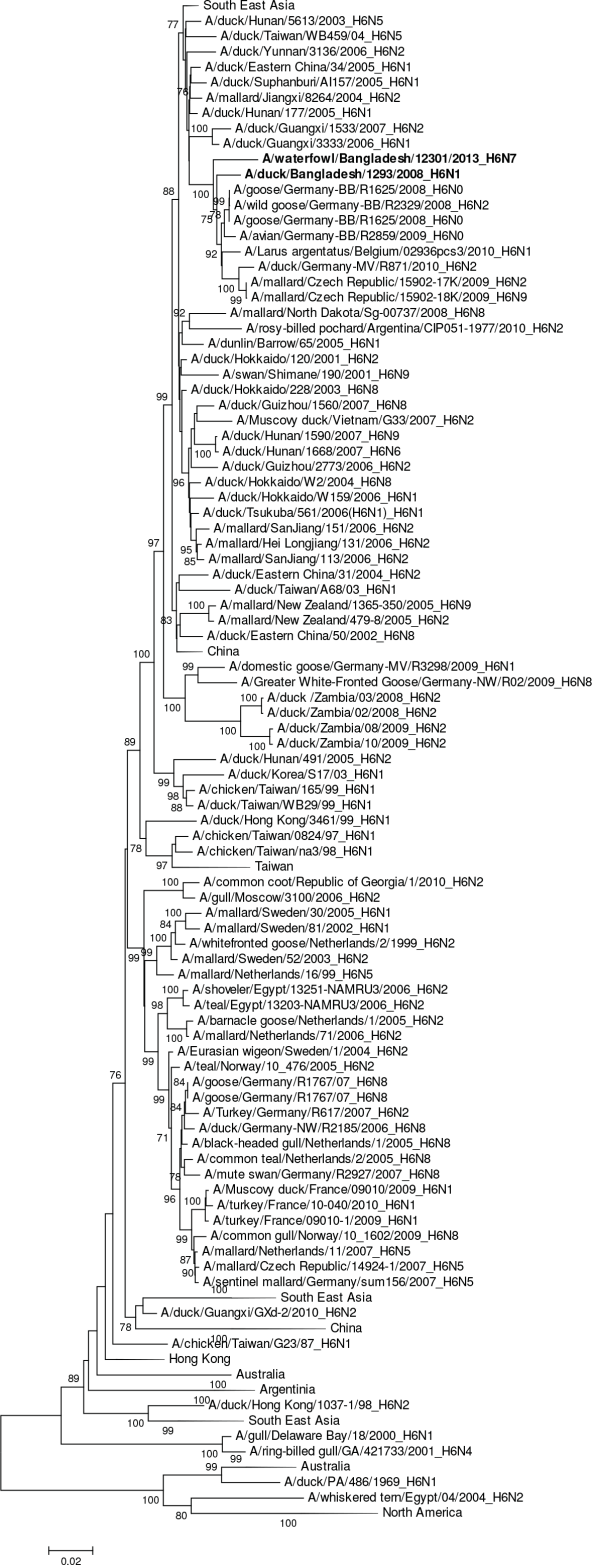
Phylogenies of the complete coding hemagglutinin genes for subtype H6. The viruses identified in this study are shown in boldface. For clarity large branches were collapsed and labeled according to the geographic location or collection years of viruses in that branch. Bootstrap values ≥70 are shown on branches.

**Fig 8:**
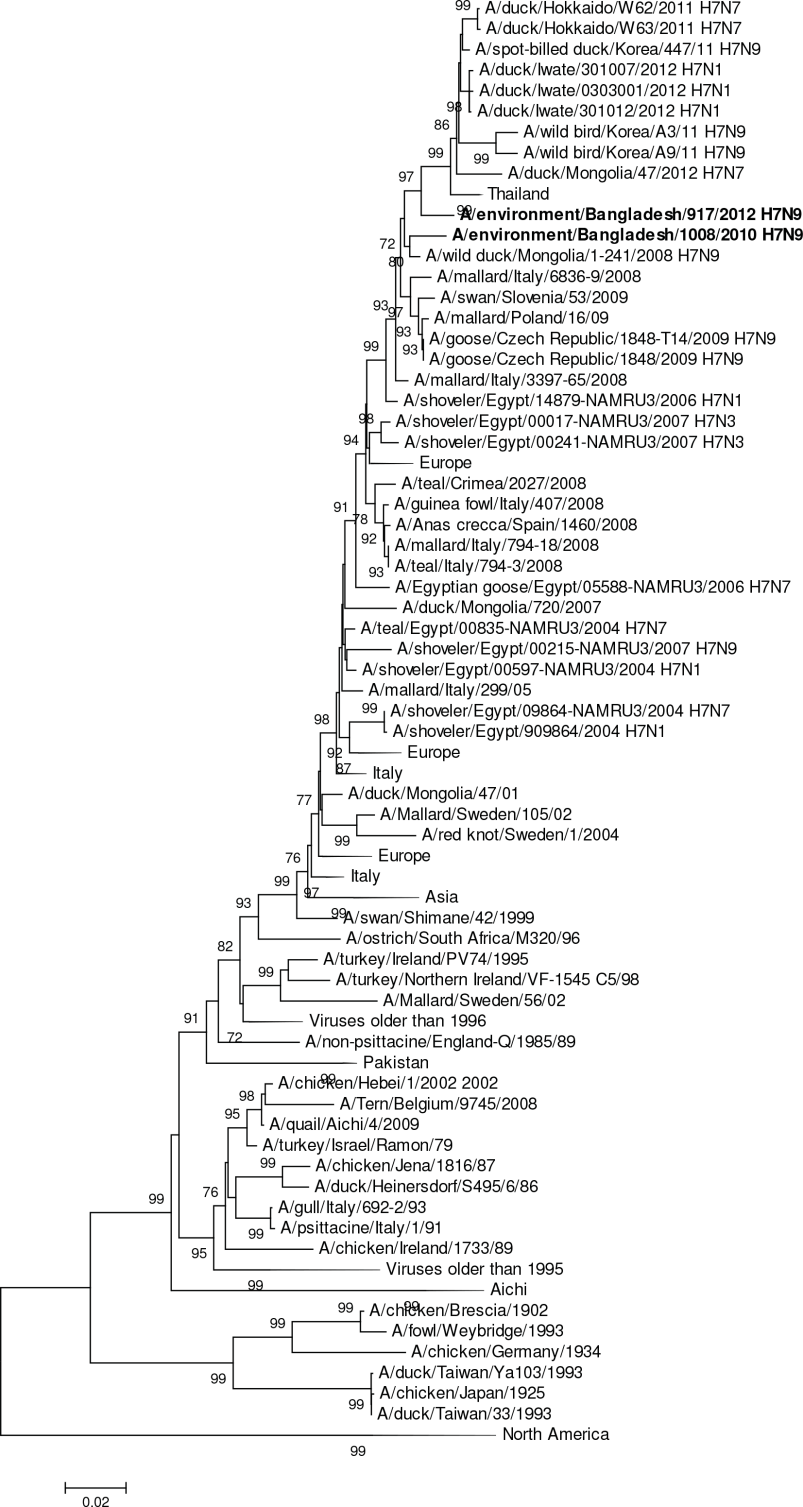
Phylogenies of the complete coding hemagglutinin genes for subtype H11. The viruses identified in this study are shown in boldface. For clarity large branches were collapsed and labeled according to the geographic location or collection years of viruses in that branch. Bootstrap values ≥70 are shown on branches.

The single H6N1 virus was genetically related to European viruses and was 98% identical by nucleotide analysis to A/goose/Germany-BB/R1625/2008 (H6) (Fig 7). It was phylogenetically unrelated to the H6N1 virus associated with human infection in Taiwan (A/Taiwan/2/2013, GISAID EPI459855) [31]. The HA gene segment of A/waterfowl/Bangladesh/12301/2013 (H6N7) was phylogenetically closely related to European viruses and the HA gene of A/duck/Bangladesh/1293/2008 (H6N1) with 97% nucleotide identity (Fig 7). The HA gene segments of the two 2007 Bangladesh H11N3 viruses were most closely related to European viruses A/mallard/Netherlands/17/2007 (H11N8) with 97% sequence identity (Table 2, Fig 8). In contrast, the H11 gene segments from avian and environmentally-derived H11N3 viruses collected in 2009 and 2010 were genetically most closely related to viruses from Japan and China (A/chicken/Nanjing/908/2009 [H11N2]) with 98% nucleotide sequence identity (Table 2, Fig 8).

### Genetic and antigenic characteristics of H7N9 and H9N2 viruses

**Fig 9:**
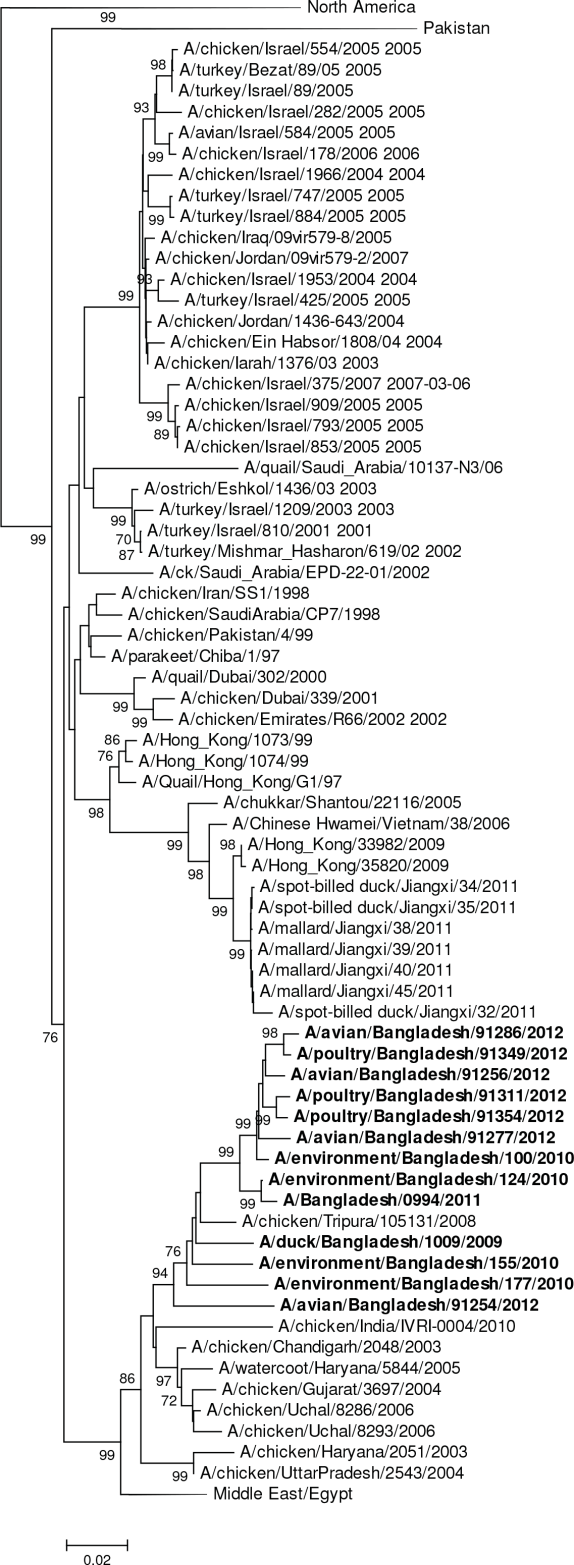
Phylogenies of the complete coding hemagglutinin genes for subtype H7. The viruses identified in this study are shown in boldface. For clarity large branches were collapsed and labeled according to the geographic location or collection years of viruses in that branch. Bootstrap values ≥70 are shown on branches.

The H7N9 virus (A/environment/Bangladesh/1008/2010) collected in 2010 was phylogenetically closer to Eurasian strains than to Southeast Asian strains of H7N9 in both the H7 and N9 genes (Fig 9, S1 Fig). The HA gene segment was phylogenetically most closely related to a Central Asian virus (A/wild duck/Mongolia/1-241/2008 [H7N9]) sharing 98% nucleotide identity (and differed by only seven amino acid residues in the HA protein. The 2012 H7N9 virus (A/environment/Bangladesh/917/2012) shared 97.3% nucleotide identity and differed by 5 amino acids in the HA protein compared to the 2010 Bangladesh strain. Both H7N9 viruses were phylogenetically unrelated to 2013–2014 Chinese H7N9 viruses (Fig 9) and were 91.4% identical in their nucleotides (21 amino acid changes in the HA protein).

Viruses that are genetically related may be antigenically related as well. That relationship can be measured by the HI assay using reference antisera. The basis of the HI assay is that antibodies to influenza virus will prevent attachment of the virus to red blood cells. Therefore hemagglutination is inhibited when antibodies are present. If viruses show similar HI titers against a reference serum they belong to the same antigenic group. If titers are more than 4 or 8 fold different from their homologous HI titers when tested to the same serum they are not considered to belong to the same antigenic group.

To determine the antigenic characteristics of one of the LPAI H7N9 viruses isolated in this study, A/environment/Bangladesh/917/2012 (H7N9) was used to produce antisera in ferrets and characterized antigenically using a panel of reference antisera raised against North American and Eurasian lineage H7 subtype viruses from avian influenza outbreaks in the US (Virginia), in Europe (Netherlands), and Asia (Vietnam and China) (Table 3). Antisera raised to the Bangladesh H7N9 virus demonstrated relatedness with viruses from the same genetic lineage, namely the Eurasian H7 viruses A/Netherlands/219/2003 (H7N7) and A/mallard/Netherlands/12/00 (H7N3), showing that these viruses are antigenically closely related (only a two–fold difference in titer when compared to the homologous virus titer). Chinese LPAI H7N9 viruses were inhibited to a lesser degree by the Bangladesh virus antisera (four-fold lower than the homologous virus titer). Antisera against A/environment/Bangladesh/917/2012 was least reactive with genetically unrelated viruses from Vietnam H7N3 (8-fold titer decrease) or North American H7N2 virus, A/turkey/Virginia/5429/02 (16-fold reduced titer, Table 3).

**Table 3:** Antigenic characterization of a H7 virus isolated from Bangladesh in 2012.

STRAIN DESIGNATION	NORTH AM*	VIETNAM	EURASIAN				
	TK/VA/4529	DK/VN/197	NL/219	ML/NL/12	ANHUI/1/13	SHANG/1/13	ENV/BA/917
A/TURKEY/VIRGINIA/4529/2002 (H7N2)	**160**	20	80	80	20	160	40
A/DUCK/VIETNAM/NCVD-197/2009 (H7N3)	20	**80**	160	160	40	80	80
A/NETHERLANDS/219/2003 (H7N7)	10	20	**160**	320	10	80	320
A/MALLARD/NETHERLANDS/12/2000 (H7N3)	20	80	320	**640**	20	320	320
A/ANHUI/1/2013 (H7N9)#	40	40	160	160	**80**	320	160
A/SHANGHAI/1/2013 (H7N9)	40	40	160	320	40	**320**	160
A/ENVIRONMENT/BANGLADESH/917/2012 (H7N9)	20	80	320	640	80	320	**640**

Hemagglutination inhibition (HAI) titers of ferret antisera to H7 viruses are shown and listed by HA clade. The homologous titer for each of the reference viruses/antisera is boldfaced and underlined. Boxes indicate genetically related HA genes

#candidate vaccine virus

*NORTH AM-North America

**Fig 10:**
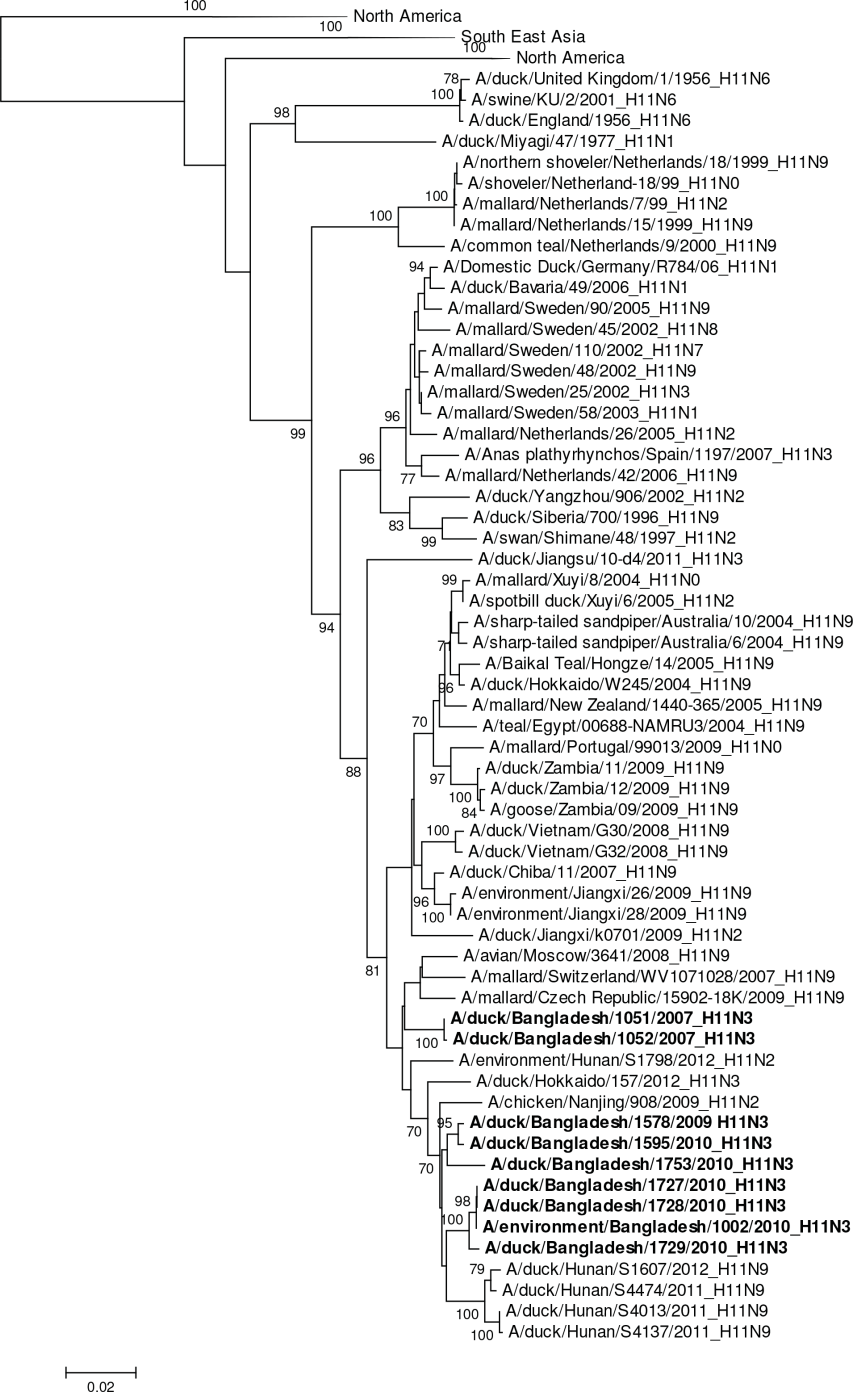
Phylogenies of the complete coding hemagglutinin genes for subtype H9. The viruses identified in this study are shown in boldface. For clarity large branches were collapsed and labeled according to the geographic location or collection years of viruses in that branch or the official lineage denominations. Bootstrap values ≥70 are shown on branches.

All 12 H9 HA gene segments were phylogenetically closely related to viruses from Bangladesh and India forming a monophyletic cluster within the larger G1 lineage of H9N2 subtyped viruses (Fig 10). The human virus (A/Bangladesh/0994/2010) differed by a single amino acid in the HA protein from the closely related avian virus (A/environment/Bangladesh/124/2010 [H9N2]). The closest genetically related virus (A/chicken/Bangladesh/VP01/2006 [H9N2]) differed at 4 amino acid changes to A/duck/Bangladesh/1009/2009 (H9N2) (or with a nucleotide difference of 1.1%). These H9N2 viruses were antigenically analyzed in comparison to representative viruses from past and contemporary lineages of H9 viruses (Tables 4 and 5). The viruses showed a high level of cross-reactivity with H9N2 viruses that belong to the G1 lineage and were collected in Bangladesh’s poultry. The reactivity of sera was highest when compared to other G1 lineage viruses collected in the country (homologous titer of sera against A/Bangladesh/0994/2011 was equivalent or within 4-fold to heterologous titers, Tables 4 and 5). Bangladesh viruses showed a greater reduction in heterologous titers against G1 lineage viruses from locations outside South East Asia (such as A/Hong Kong/1073/1999, A/quail/Hong Kong/G1/1997 and A/Hong Kong/33982/2009). This indicates that these viruses had drifted antigenically from that group (heterologous HI titers on average 32 to 64-fold reduced). We did not observe any antigenic relationship of A/Bangladesh/0994/2011 H9N2 antisera with viruses of distinct antigenic H9 lineages, such as Y280-like, CK-BEI-like or BY280-like, (Tables 4 and 5).

**Table 4:** Antigenic characterization of H9N2 viruses isolated from Bangladesh (2009 through 2011).

	REFERENCE FERRET ANTISERA										
	VN LINEAGE	KOR	G1 LINEAGE				A Y280-LIKE (G9)		CK-BEI-LIKE	B Y280-LIKE	
STRAIN DESIGNATION	DK/VN/227	CK/KO	HK/ 1073	QL/HK/G1	HK/ 33982	BA/0994	LBM/HK/1226	CK/HK/G9	HK/2108	HK/3239	DK/HK
A/DUCK/VIETNAM/NCVD-227/2009	**80**	5	5	5	5	10	10	10	5	5	5
A/CHICKEN/KOREA/96323/1996	20	**640**	5	5	5	20	20	20	5	10	5
A/HONG KONG/1073/1999#	5	5	**320**	320	320	20	5	20	5	5	5
A/QUAIL/HONG KONG/G1/1997	5	5	320	**640**	320	80	5	20	5	5	5
A/HONG KONG/33982/2009#	10	5	320	160	**1280**	160	20	80	10	5	5
A/BANGLADESH/0994/2011#	80	80	160	80	320	**5120**	640	1280	640	640	320
A/LBM/HONG KONG/1226/1999	80	5	40	80	80	20	**320**	1280	320	320	320
A/CHICKEN/HONG KONG/G9/1997	320	80	80	640	640	2560	2560	**5120**	1280	1280	1280
A/HONG KONG/2108/2003	160	5	20	5	80	160	160	320	**1280**	80	160
A/HONG KONG/3239/2008	320	160	40	160	160	640	640	1280	640	**640**	640
A/DUCK/HONG KONG/Y280/1997	160	80	5	80	20	320	320	640	320	320	**640**
**TEST ANTIGENS**											
A/DUCK/BANGLADESH/1009/2009	40	40	80	40	80	2560	320	640	160	160	160
A/ENVIRONMENT/BANGLADESH/124/2010	20	40	40	40	80	1280	160	320	160	80	80
A/ENVIRONMENT/BANGLADESH/155/2010	40	80	80	40	160	5120	320	640	320	320	320
A/ENVIRONMENT/BANGLADESH/177/2010	40	80	80	40	160	1280	320	640	160	320	160

Hemagglutination inhibition (HAI) titers of ferret antisera to A(H9N2) viruses are shown and listed by HA clade. The homologous titer for each of the reference viruses/antisera is boldfaced and underlined. Boxes indicate genetically related HA genes

#candidate vaccine viruses, VN = Vietnam, CK-BEI-Chicken/Beijing

**Table 5:** Antigenic characterization of H9N2 viruses isolated from Bangladesh (2012 through 2013).

	REFERENCE FERRET ANTISERA									
	VN LINEAGE	KOREAN	G1 GROUP				A Y280-LIKE (G9)	CK-BEI-LIKE	B Y280-LIKE	
STRAIN DESIGNATION	DK/VN/227	CK/KO	HK/1073	QL/HK/G1	HK/33982	BA/0994	LBM/HK/1226	HK/2108	HK/3239	DK/HK
A/DUCK/VIETNAM/NCVD-227/2009	**160**	160	5	5	5	160	40	40	10	5
A/CHICKEN/KOREA/96323/1996	10	**160**	5	5	5	5	5	5	5	5
A/HONG KONG/1073/1999#	5	5	**320**	640	320	40	5	5	5	5
A/QUAIL/HONG KONG/G1/1997	5	5	160	**640**	160	80	5	5	5	5
A/HONG KONG/33982/2009#	10	5	160	160	**640**	80	20	5	5	5
A/BANGLADESH/0994/2011#	80	160	160	160	320	**5120**	640	640	320	640
A/LBM/HONG KONG/1226/1999	160	80	20	160	20	320	**640**	320	320	640
A/HONG KONG/2108/2003	40	5	5	80	40	160	80	**1280**	80	160
A/HONG KONG/3239/2008	160	160	20	80	20	640	640	640	**640**	2560
A/DUCK/HONG KONG/Y280/1997	80	20	5	40	5	160	320	320	160	**1280**
**TEST ANTIGENS**										
A/AVIAN/BANGLADESH/91256/2012	10	40	80	40	80	2560	320	320	160	640
A/AVIAN/BANGLADESH/91254/2012	10	40	80	40	80	5120	320	320	160	640
A/AVIAN/BANGLADESH/91277/2012	10	40	40	40	40	5120	320	160	80	640
A/AVIAN/BANGLADESH/91286/2012	10	20	40	40	40	5120	160	160	80	320
A/POULTRY/BANGLADESH/91311/2012	10	40	80	40	40	5120	320	320	160	640
A/POULTRY/BANGLADESH/91349/2012	20	40	80	80	80	2560	320	320	160	640
A/POULTRY/BANGLADESH/91353/2012	20	40	80	80	40	5120	320	320	160	640
A/POULTRY/BANGLADESH/91354/2012	20	80	80	80	80	5120	320	320	160	320
A/POULTRY/BANGLADESH/91412/2013	5	20	40	10	40	2560	160	160	80	320

Hemagglutination inhibition (HAI) titers of ferret antisera to A(H9N2) viruses are shown and listed by HA clade. The homologous titer for each of the reference viruses/antisera is boldfaced and underlined. Boxes indicate genetically related HA genes

#candidate vaccine viruses

VN = Vietnam, CK-BEI-Chicken/Beijing

### Phylogeny of NA genes (N1 through N9)

Phylogenies and nucleotide differences were analyzed individually for neuraminidase gene segments subtype N1 through N9. Five N1 NA gene segments from H1N1 and H6N1 viruses were phylogenetically closely related and formed a group with Eurasian (e.g., A/mallard/Republic of Georgia/1/2010 [H10N1]) and South East Asian sequences (A/duck/Hokkaido/W26/2012 [H12N1], S1 Fig). The N1 gene segments were genetically distinct from the N1 found in highly pathogenic avian influenza A(H5N1) viruses. Swine and seasonal influenza N1 gene segments formed a distinct phylogenetic group unrelated to avian viruses (S1 Fig). The NA gene segment of the single H6N1 virus (A/duck/Bangladesh/1293/2008) had 98% nucleotide identity to a geographically proximal virus (A/aquatic bird/India/NIV-17095/2007 [H11N1]).

The N2 NA gene segment nucleotide comparison was split into separate analyses due to the large number of available sequences and genetic differences of the non-H9N2 LPAI viruses (S1 Fig) and H9N2 virus nucleotide sequences (S1 Fig). The N2 sequences of Bangladesh H5N2 and H4N2 viruses were phylogenetically closely related to N2 found in Asia (e.g. A/baikal teal/Xianghai/426/2011 [H5N2], S1 Fig). The three H3N2 viruses grouped separately with Central Asian (A/gadwall/Altai/1202/2007 [H5N2]) and South East Asian viruses and were distinct from the other Bangladesh viruses (S1 Fig). The NA of seasonal H3N2 viruses, swine H1N2 and H3N2 viruses were genetically diverse and did not cluster with avian HxN2 NA genes (data not shown). All H9N2 N2 gene segments formed a monophyletic group with Indian and other Bangladeshi H9N2 viruses (S1 Fig).

The NA’s of two H11N3 viruses were phylogenetically most closely related to European viruses (e.g. Italy, Germany), while the remaining eight H11N3 viruses were genetically more closely related to N3 containing viruses circulating in South East Asia (S1 Fig). The three N4 gene segments formed a common phylogenetic cluster with viruses from Thailand and Eurasian countries (S1 Fig). Nucleotide similarity between the closest related virus was 98.7% (A/duck/Thailand/CU-9744C/2010 [H7N4]). The N6 gene segment was phylogenetically separated into two groups with most of the N6 gene segments clustering with Eurasian viruses (e.g. A/common pochard/Aktau/1455/2006 [H4N6]) along with a virus from Pakistan (A/Khaki Campbell duck/Karachi/NARC-23963/2010 [H4N6]) (S1 Fig). The second N6 genetic group was composed of two identical N6 NAs, which were most closely related to N6 NAs found in viruses from South East Asia (e.g. A/Muscovy duck/Thailand/CU-LM1983/2009 [H4N6]) (S1 Fig). The NA of A/waterfowl/Bangladesh/12301/2013 (H6N7) genetically clustered apart from larger monophyletic group of viruses containing South East Asian H7N7 viruses (S1 Fig). The most closely related virus identified was a Japanese strain (A/duck/Tsukuba/30/2007 (H7N7)), which had a nucleotide similarity of 95%.

The three N8 gene segments from H3N8 viruses 100% nucleotide identity and were genetically closely related (99% nucleotide identity) to Asian viruses such as A/wild duck/Jiangxi/19615/2009 (H7N8) (S1 Fig). The N9 NA of subtype H7N9 (A/environment/Bangladesh/1008/2010) phylogenetically clustered closely with European and African viruses (e.g. A/goose/Czech Republic/1848-T14/2009 [H7N9], A/shoveler/Egypt/00004-NAMRU3/2007 [H10N9], S1 Fig). This virus differed by 3.1% at the nucleotide level compared to Chinese H7N9 viruses. The other N9 gene derived from A/environment/Bangladesh/917/2012 (H7N9) was similar to viruses from South East Asia, Europe and Australia collected between 2005 and 2010 (S1 Fig). The Bangladeshi H7N9 viruses showed diversification with 11 amino acid changes in their N9 NA proteins.

### Genotyping of internal genes

**Fig 11:**
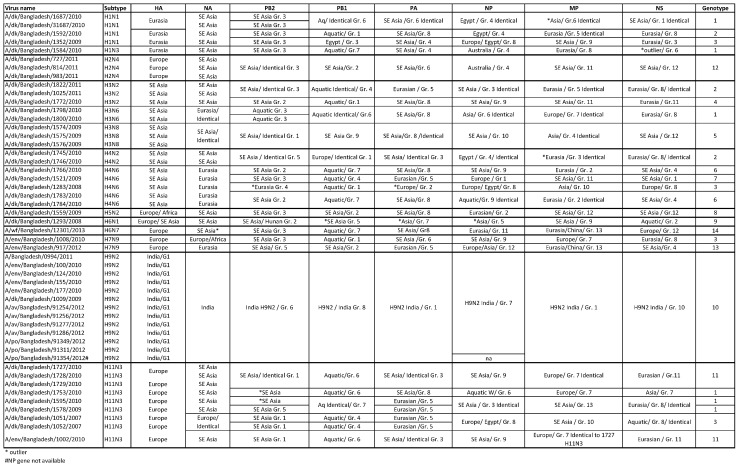
Table lists genotypes of viruses from this study by strain name, subtype, and phylogenetic relationship. Identical viruses were merged for clarity when applicable. The group numbering was based on the individual complete coding gene analysis. Abbreviations: dk–duck, env–environment, SE–South East, Gr–group.

To assess genotypic diversity and potential reassortment between HPAI (H5N1) and LPAI viruses, gene sequences of H5N1 viruses were analyzed in large alignments that were used for tree reconstruction. Based on phylogenetic clustering of internal genes, the 50 virus genomes could be divided into 14 distinct genotypes (Fig 11). Most internal protein coding gene constellation genotypes were composed of mixed subtypes. Genotype 1, for instance, contained eight viruses with different subtypes H1N1, H1N3, H3N6, and H11N3 and these were collected from different sites in Bangladesh (Fig 1, Table 2, and Fig 11). The H11N3 viruses that shared genotype 1 were collected between December 2009 and September 2010 at different times but in the same location. Genotypes 4, 7, 8, and 9 contained H3N2 (A/duck/Bangladesh/1772/2010), H4N6 (A/duck/Bangladesh/1521/2009) and the only H5N2 and H6N1 viruses (Fig 11). Three H4N6 viruses were collected from the same site on different dates and differed in their genotypes. Genotype 11 contained H11N3 viruses sampled in the northwestern region and from southeastern Chittagong Division (Table 2, Fig 11). Few viruses clustered outside of a common group in the PB2 gene, and were further apart from ancestral virus sequences indicated by a longer branch length (i.e., A/duck/Bangladesh/1283/2008 (H4N6) or A/duck/Bangladesh/1595/2010 [(H11N3]) (Fig 11).

All subtype H9N2 viruses were unified in genotype 10; a group that lacked viruses with other subtypes (Fig 11). The viruses from bird, environmental and human hosts all interleaved within phylogenetic trees containing H9N2 viruses from Bangladesh and India for each of the six internal protein coding gene segments independent of their collection year or the species they were collected from (S2 Fig). All H9N2 viruses formed a monophyletic group with A/chicken/Tripura/105131/2008 (H9N2) (S2 Fig).

In phylogenetic trees of the PB2, PB1 and PA genes, Bangladeshi nucleotide sequences grouped mostly with Asian, Eurasian, Northern African or European viruses with no distinct geographic or subtype-related pattern (S2 Fig). In their NP gene segments, the three H3N8 viruses formed a cluster with older Chinese sequences (collected between in 2004–2005) with 1–2 amino acid changes (S2 Fig). H1N1, H1N3 and H4N2 viruses were genetically more closely related to Australian H11N9 viruses from 2004 than to more recent viruses in the region (S2 Fig). A single virus, A/duck/Bangladesh/1521/2009 (H4N6), shared common ancestors with Central Asian H9N2 viruses with 96% nucleotide identity. All other viruses clustered with South East Asian viruses (S2 Fig). The M gene sequences clustered mainly with Asian, European, or Eurasian viruses (S2 Fig). Two viruses of subtype H4N6 (A/duck/Bangladesh/1766/2010; A/duck/Bangladesh/1783/2010) were more closely related to a virus from Vietnam (A/duck/Viet Nam/LBM300/2012 [H10N2]) with 98% nucleotide sequence identity when compared to either of these viruses (S2 Fig). Most viruses described herein grouped with allele A viruses in their NS genes (S2 Fig). Asian, European and African viruses were genetically very close with other Bangladesh viruses forming a monophyletic group. The six internal protein coding gene segments of both H7N9 viruses (A/environment/Bangladesh/1008/2010 and A/environment/Bangladesh/917/2012) were similar to other viruses from neighboring countries (S2 Fig). Phylogenetic analysis indicates that the internal protein coding genes of these Bangladesh H7N9 viruses share common ancestors with Eurasian lineage LPAI viruses from various hosts, but differed from Chinese H7N9 viruses from 2013 and 2014, some of which have been transmitted to humans.

## Discussion

We screened viruses collected through multi-year surveillance or systematic outbreak investigations in live bird markets and backyard flocks in Bangladesh for the presence of influenza A viruses. In our study we characterized the distribution of multiple subtypes of LPAI viruses and the genetic and antigenic features of viruses isolated. Fifty coding-complete genome sequences of LPAI viruses were compared to previously identified viruses revealing 14 different subtypes of low pathogenicity avian influenza viruses and 14 discrete genotypes. The characterization of their genomes indicated that the majority of the viruses were related to viruses found in South East Asia, Europe, and Africa. Surface and internal protein coding gene segments were distributed over diverse geographic locations within Bangladesh and viruses generally grouped regardless of their subtype with the exception of H9N2 viruses that formed a unique monophyletic group for each gene. In this study, none of the internal protein coding gene segment nucleotide sequences were closely related to enzootic HPAI A(H5N1) viruses [13, 15].

A large percentage of specimens tested, were positive for avian influenza A virus by real-time RT-PCR. The majority of the viruses were H5 and H9 negative. Approximately half of the HPAI H5 positive samples and seven H9N2 LPAI viruses were collected during poultry outbreak investigations reconfirming the enzootic circulation of H5 and H9 subtypes in poultry in the country [32]. This study focused on non-HPAI H5N1 viruses in order to better understand the ecology, distribution and diversity of LPAI subtypes in the country. H9N2, H11N3, and H4N6 subtypes were detected most frequently, while H1N1, H2N4, H3N2, H3N8, and H4N2 subtypes were found in smaller numbers.

The HA and NA gene segments of each individual subtype were often phylogenetically most closely related to other viruses collected from Bangladesh, suggesting persistence of specific LPAI subtypes in Bangladesh and illustrating that there is very limited nucleotide sequence data available from closely related viruses. The viruses characterized here shared common ancestors with LPAI viruses from Europe, Africa and Central Asia indicating possible transient introductions of LPAI viruses as well. Most viruses did not form monophyletic groups with viruses of the same subtype. The exception was two H3N2 and two H4N6 viruses that were collected on the same day and from the same location. In addition, all 12 H9N2 virus sequences (including one human-origin H9N2 virus) formed a monophyletic group with contemporary H9N2 viruses found in Bangladesh and in India in all eight of their gene segments.

A major role of pandemic preparedness is the assessment of genetic and antigenic relationships of circulating LPAI viruses with the potential to infect humans. In our study, an H5N2 virus differed significantly from H5 viruses that exhibit high pathogenicity in birds or were previously found in humans [4]. Two H7N9 viruses were phylogenetically and antigenically unrelated to the human and avian H7N9 viruses identified in China in 2013–2015. Furthermore, they did not possess the molecular markers described in Chinese LPAI H7N9 viruses believed to facilitate host cell receptor binding and replication in humans suggesting they pose little risk to humans [5, 33–35]. Antigenically, the Bangladesh H7N9 virus was well-inhibited in HI assays by ferret antisera produced against other Eurasian lineage H7 subtype viruses and the nearest WHO candidate vaccine virus, A/mallard/Netherlands/12/2000, showing that previously developed vaccine candidates targeting this lineage would likely protect against H7N9 viruses in Bangladesh. Both HA genes of the H6 subtype viruses were unrelated to an H6N1 virus discovered in a sick patient in Taiwan [36, 37]. Interestingly, H9N2 viruses detected in Bangladesh follow a pattern similar to those found in other countries were this subtype is enzootic in poultry. Likely due to persistent circulation with few re-introductions from outside regions, these viruses evolve separately from other subtypes. As has been noted previously, the evolutionary trajectory of H9N2 viruses is more akin to that described for the HPAI A(H5N1) viruses found in various foci of endemicity [38]. The ongoing evolution of H9 viruses in Bangladesh is most probably driven by frequent waves of H9N2 infection among the high density poultry populations in the country, where mitigation is complicated by a lack of overt clinical symptoms in infected flocks [32, 39]. For pandemic preparedness planning a continuous update of an H9N2 subtype candidate vaccine virus is required [32, 40, 41]. As evidenced by HI testing performed herein, the H9N2 viruses circulating in Bangladesh remain antigenically well-matched with an existing WHO candidate vaccine virus, A/Bangladesh/0994/2011. Generally, in human cases, the associated disease symptoms have been mild and there has been no evidence of human-to-human transmission [32]. The complete genome sequence of an H9N2 virus isolated from a patient in Bangladesh (A/Bangladesh/0994/2011) displayed common ancestry with LPAI A(H9N2) viruses that were enzootic in the country or neighboring countries, such as India, at the time of collection. Given that H9N2 viruses were consistently found from 2009 through 2013, suggests continuous circulation in Bangladesh’s live bird markets and ample opportunity to infect humans. Genetically and antigenically, the human H9N2 differed little when compared to its avian H9N2 counterparts described herein suggesting this subtype possesses some features associated with moderate risk to human health and should be monitored as a potential source of human illness.

This study shows that overall diverse genotypes were geographically distributed across Bangladesh, regardless of virus subtype, or host species. Viruses that were collected on identical dates and locations shared the same subtype and identical internal protein coding genes indicating a sampling effect. Three viruses classified as genotype 1 shared matching subtypes (H11N3) and locations (Netrokona), but were collected in different years (Fig 4). That genotype might have circulated continuously in the Netrokona area throughout 2007 and 2010. Genotypes 8 and 9 were represented by a single virus of a specific subtype, such as H3N6 and H4N6.These examples may indicate internal protein coding gene flow in ecologically linked viruses of the same subtype or genetic compatibilities that certain subtypes possess.

Avian influenza viruses travel either in their natural hosts (aquatic wild birds) or through poultry trade within the region, similar to what has been described for the spread of HPAI A(H5N1) viruses in South East Asia [42]. Several viruses that shared the same genotype were found in geographically dispersed regions and collected at different times indicating that some LPAI viruses in Bangladesh circulate continuously in the country and may be dispersed either via human-driven poultry movement or wild birds. We found a mix of LPAI subtypes in poultry and identified only one subtype (H2N4) in ducks grazing in the vicinity of wild birds, suggesting that poultry serve as a potential source for virus spread. As the study focused on samples primarily collected from live bird markets and outbreaks, it is impossible to know if wild birds might also contribute to this distribution. In light of these findings, temporary closures of live bird markets, especially in high poultry density districts, could pose an effective tool to combat avian influenza transmission if applied in Bangladesh [43]. Although our data indicated extensive reassortment among LPAI viruses when comparing the internal genes, there were no signs of reassortment between previously identified genomes of HPAI H5N1 and the LPAI viruses described in this study. Although HPAI H5N1 and H9N2 viruses in Bangladesh have reassorted in the past likely from long term co-circulation in domestic poultry, lack of endemicity of these LPAI viruses in poultry may produce fewer opportunities for reassortment with either H5N1 or H9N2 viruses [13]. It is also possible that ducks infected with LPAI viruses described herein might be protected from infection with HPAI H5N1 viruses preventing reassortment opportunities [44].

In conclusion, we found that low pathogenicity and highly pathogenic avian influenza viruses occur frequently in poultry sampled in live bird markets in Bangladesh, which reflects parts of previous studies [13, 15]. However, these studies looked at a single subtype. Here we present a comprehensive dataset with 14 different LPAI subtypes including 50 complete protein coding sequences in comparison to up to 5300 available virus sequences from public databases. Additionally, we showed for the first time genetic combined with antigenic properties of LPAI viruses that had infected humans in the past and are of public health importance. In Bangladesh, domestic poultry sold in live bird markets carry a wide range of LPAI virus subtypes and a high diversity of genotypes over a sampling period of seven years. Moreover, they carry LPAI viruses that are known to infect humans, such as H9N2, and other viruses related to subtypes with zoonotic potential (i.e., H6N1, or H7N9). While much remains unknown about the potential for other LPAI subtypes to infect humans, surveillance for LPAI viruses in live bird markets, and outbreak settings serves as an effective tool to detect the major AIVs that pose a public health risk and lays a foundation for both veterinary and public health officials in the event that one or more of these viruses causes outbreaks or significant disease in animals or humans.

## Material and Methods

### Ethics Statement

Bird sampling methods were reviewed and approved by the Institutional Animal Care and Use Committee. The animals sampled for this study did not include endangered or protected species. The sampling of birds was approved by the Department of Livestock Services, Ministry of Fisheries and Livestock, Dhaka in Bangladesh and carried out on public land. All animal experiments were approved by the Centers for Disease Control and Prevention's Institutional Animal Care and Use Committee and conducted in an Association for Assessment and Accreditation of Laboratory Animal Care International-accredited animal facility.

### Sample collection

Avian influenza surveillance and outbreak investigations were conducted by the International Centre for Diarrheal Disease Research, Bangladesh (icddr,b) and various government partners (Institute for Epidemiology, Disease Control and Research; Department of Livestock Services; Department of Forestry) as well as international partners (CDC; EcoHealth Alliance) from 2007 through 2013. These projects included active live bird market surveillance and specimens collected from bird carcasses, or from live birds trapped during outbreak investigations as previously described [13]. The majority of the live bird market surveillance sites established by icddr,b are located in Dhaka city. Individual fecal, cloacal, oropharyngeal or tracheal swabs were collected from birds sold at markets or kept in backyards. In live bird markets, environmental swabs were sampled from bird droppings and surfaces used for processing birds. A previously described human H9N2 virus isolated from a nasopharyngeal wash specimen collected in 2011 during longitudinal population-based surveillance for respiratory disease in Dhaka was also included in the analysis [virus name: A/Bangladesh/0994/2011, subtype H9N2] [11, 23, 45, 46]. The geographic distribution of samples was mapped using coordinates of the collection locations and the boundary map of Bangladesh in ArcGIS 9.3 (Environmental System Research Institute, Redlands, CA, USA).

### Virus isolation, subtype detection and full genome sequencing

Specimens were initially screened at icddr,b for influenza A virus using a real-time reverse transcription PCR (rRT-PCR) detection kit targeting the matrix (M) gene and H5 HA gene (CDC, 2013). Aliquots of influenza A virus positive samples and a subset of untested samples that were of special interest to a specific outbreak or market investigation were sent to the Influenza Division, CDC for confirmatory testing, subtyping and further characterization. At CDC, RNA extracts from original specimens were screened again for the presence of influenza A virus, H5 subtype and additionally for H9 subtype, and NDV as described [47, 48]. We selected viruses for inoculation if they had cycle threshold (ct) values below 32 or if the sample was of particular interest to a specific outbreak or market investigation. This set of influenza A virus-positive/unsubtypable/NDV negative samples were inoculated into 10–11 day old embryonated chicken eggs and allantoic fluid was harvested 48 hours post-inoculation. Many of the influenza A-positive, unsubtypable samples had high real-time RT-PCR ct values, and virus was not isolated from these samples. Isolation-positive samples were identified by hemagglutination assay using turkey red blood cells. All infectious materials were maintained in biosafety level 3 containment, including enhancements required by the U.S. Department of Agriculture and the Select Agents program (http://www.cdc.gov/od/ohs/biosfty/bmbl5/bmbl5toc.htm). Specimens that were successfully propagated in ECEs were included in this analysis only if they were negative for the multibasic cleavage site found in HPAI H5N1 virus determined by sequence comparison of their HA gene. Genomic RNA was extracted from virus-infected allantoic fluid using the RNeasy extraction kit (Qiagen, Valencia, CA) and used as template for generation of cDNA by random hexamer-primed reverse transcription. Subtyping was performed using a multiplex PCR assay previously described [49]. Sequences of HA and NA fragments were analyzed using the *basic local alignment search tool* (BLAST) analysis against the GenBank database to predict the influenza A virus subtype [50]. The surface and internal protein genes were then amplified using subtype-specific influenza A virus primers as overlapping fragments with the Access Quick one-step RT-PCR kit (Promega, Madison, WI) and sequenced on an automated Applied Biosystems 3730 system using cycle sequencing dye terminator chemistry (ThermoFisher Scientific, Carlsbad, CA). Contigs of full length open reading frames were generated for each gene and quality of sequences were verified using Sequencher version 5.0 (Gene Codes, Ann Arbor, MI). Gene sequences were submitted to GISAID (http://platform.gisaid.org) prior to publication (Accession Nos.:EPI448288-95, EPI448280-87, EPI457484-91, EPI540152-507, EPI484574-77, EPI484579-80, EPI540527-44).

### Molecular characterization

Individual datasets contained a representative selection of publicly available HA and NA sequences for subtype-specific phylogenetic comparison of each of the eight gene segments (http://platform.gisaid.org). The internal gene analysis was performed with available influenza A sequences from avian and human hosts or the environment by searching for all subtypes. After initial assessment, unrelated seasonal viruses and non-avian virus sequences were excluded from the analysis. The searches resulted in 4,368 polymerase basic protein 2 (PB2), 4,747 polymerase basic protein 1 (PB1), 4,860 polymerase acidic protein (PA), 5,095 nucleoprotein (NP), 5,495 matrix protein (M) and 5,302 nonstructural protein (NS) gene sequences that were included. To assess potential reassortment between current HPAI H5N1 and LPAI viruses, gene sequences of all available H5N1 viruses were included also. Sequence alignments were generated with the MUSCLE algorithm incorporated in BioEdit version 5 [51, 52]. All sequence alignments were edited manually for frameshifts, sequence duplication and length (i.e., only sequences with at least 90% of the coding region were used). Nucleotide difference calculations and tree inferences were performed in MEGA5 [53]. For the external genes trees were inferred using the neighbor joining method with 1000 bootstrap replicates and we defined clusters that shared common nodes in the tree with more or equal than 70% bootstrap support. For the internal gene analysis trees were reconstructed with the Neighbor-joining method, and nucleotide differences were calculated in MEGA5. We defined a genotype using a combination of the phylogenetic relation between sequences that shared common nodes in the tree and had at least 98% nucleotide similarity.

### Antigenic characterization

To detect possible antigenic drift variants compared to pre-pandemic candidate vaccine viruses, the H7N9 and H9N2 subtypes detected were tested by hemagglutination inhibition (HI) assay with ferret antisera raised against representative H7 (H7N2, H7N3, H7N7, and H7N9) and H9 viruses (H9N2, H9N3), including WHO candidate vaccine viruses. Antiserum was also raised to the select viruses collected during this study. As previously described, viruses were standardized to 8 hemagglutination inhibition units/50μl and added to serially diluted, receptor destroying enzyme-treated, red blood cell adsorbed antisera (DENKA SEIKEN, Campbell) [54]. The HI titers were reported as the reciprocal of the last dilution of antiserum that completely inhibited hemagglutination.

## Supporting Information

S1 Fig Phylogenies of the complete coding neuraminidase genes for subtypes N1 (A), N2 (B), N2 of H9N2 viruses (C), N3 (D), N4 (E), N6 (F), N7 (G), N8 (H) and N9 (I). The viruses identified in this study are shown in boldface. For clarity large branches were collapsed and labeled according to the geographic location or collection years of viruses in that branch. Bootstrap values ≥70 are shown on branches.
(PDF)

S2 Fig Phylogenies of complete coding internal genes (A) polymerase basic 2 (PB2), (B) PB1, (C) polymerase acid (PA), (D) nucleoprotein (NP), (E) matrix protein (M), and (F) nonstructural proteins (NS). The viruses identified in this study are shown in boldface. For readability, strain names were removed for viruses that did not cluster with Bangladesh strains and/or large branches were collapsed and labeled according to the geographic location of viruses in that branch.
(PDF)
