# Supplementary figures and images for: Genetically Diverse Low Pathogenicity Avian Influenza A Virus Subtypes Co-Circulate among Poultry in Bangladesh
Source: PLoS One. 2016 Mar 24;11(3):e0152131. doi: 10.1371/journal.pone.0152131 (PMC4806916; doi:10.1371/journal.pone.0152131)

S1 Fig

A

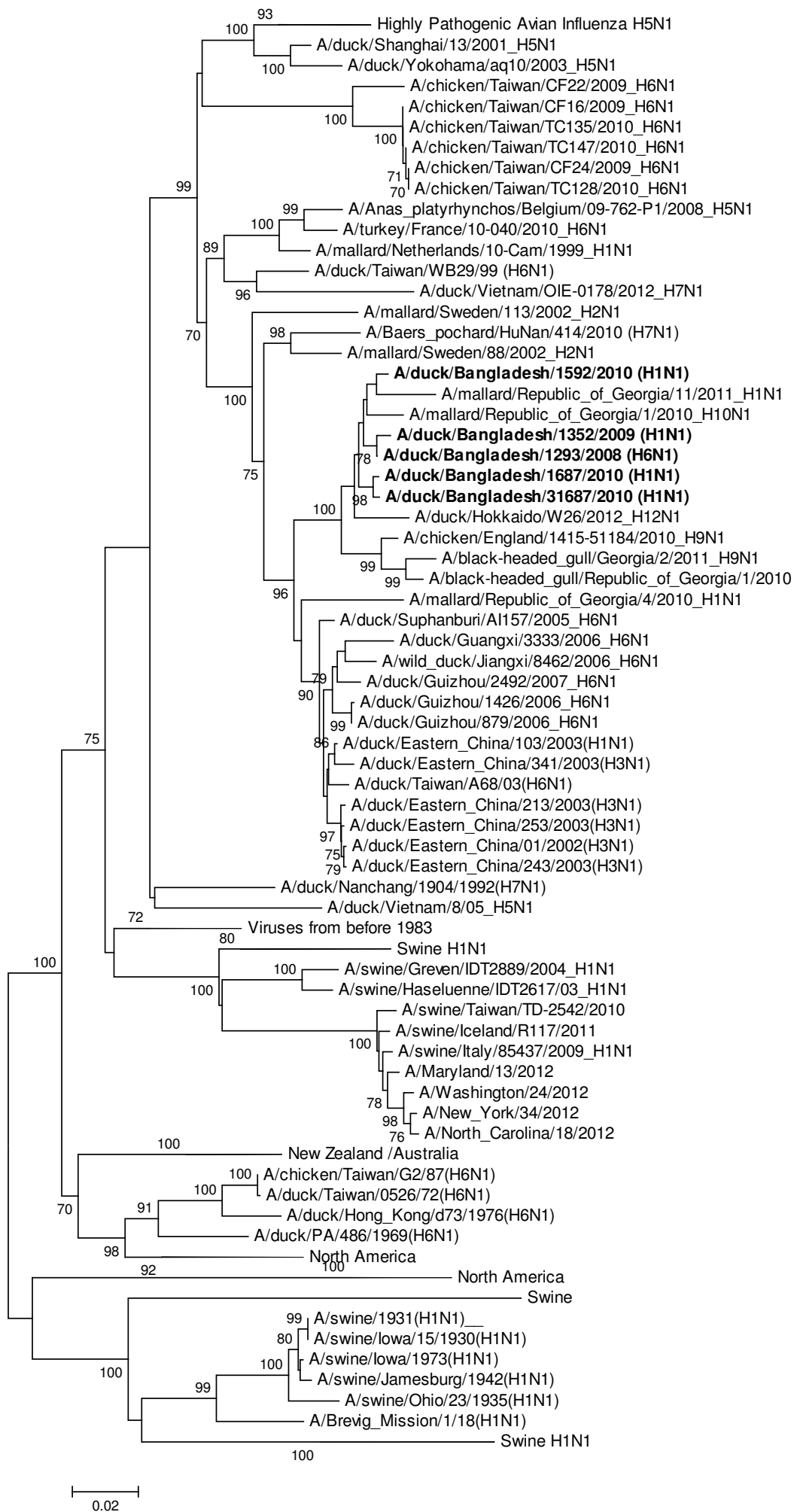

B

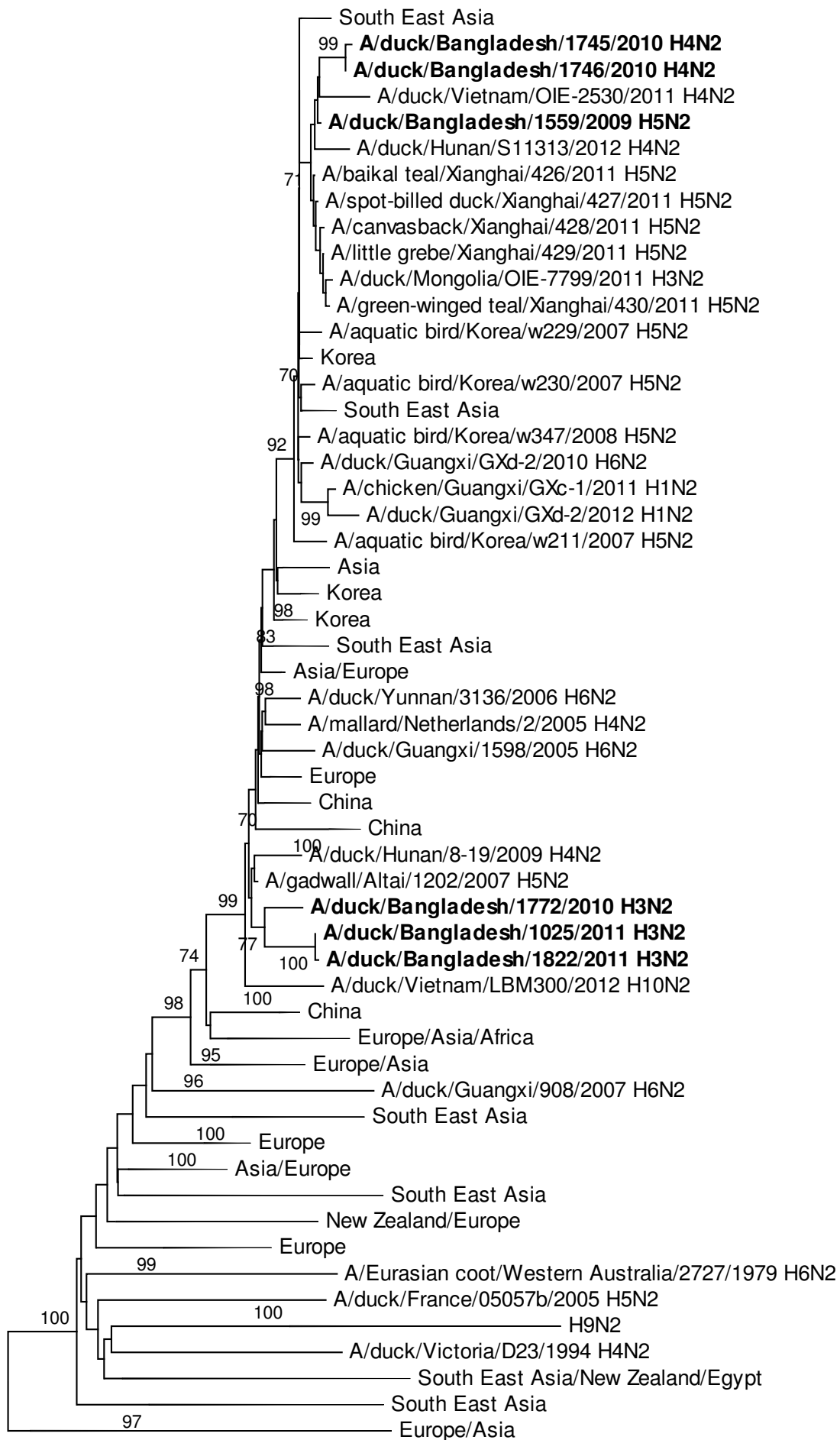

C

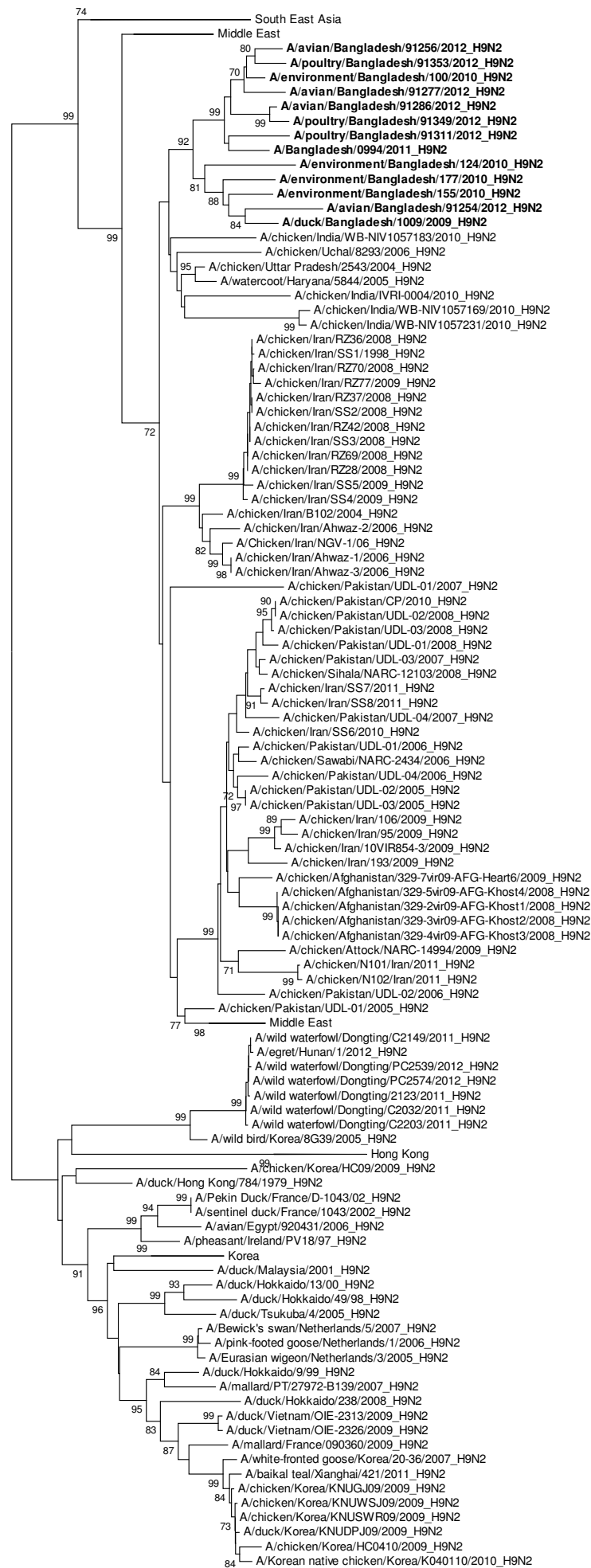

0.02

D

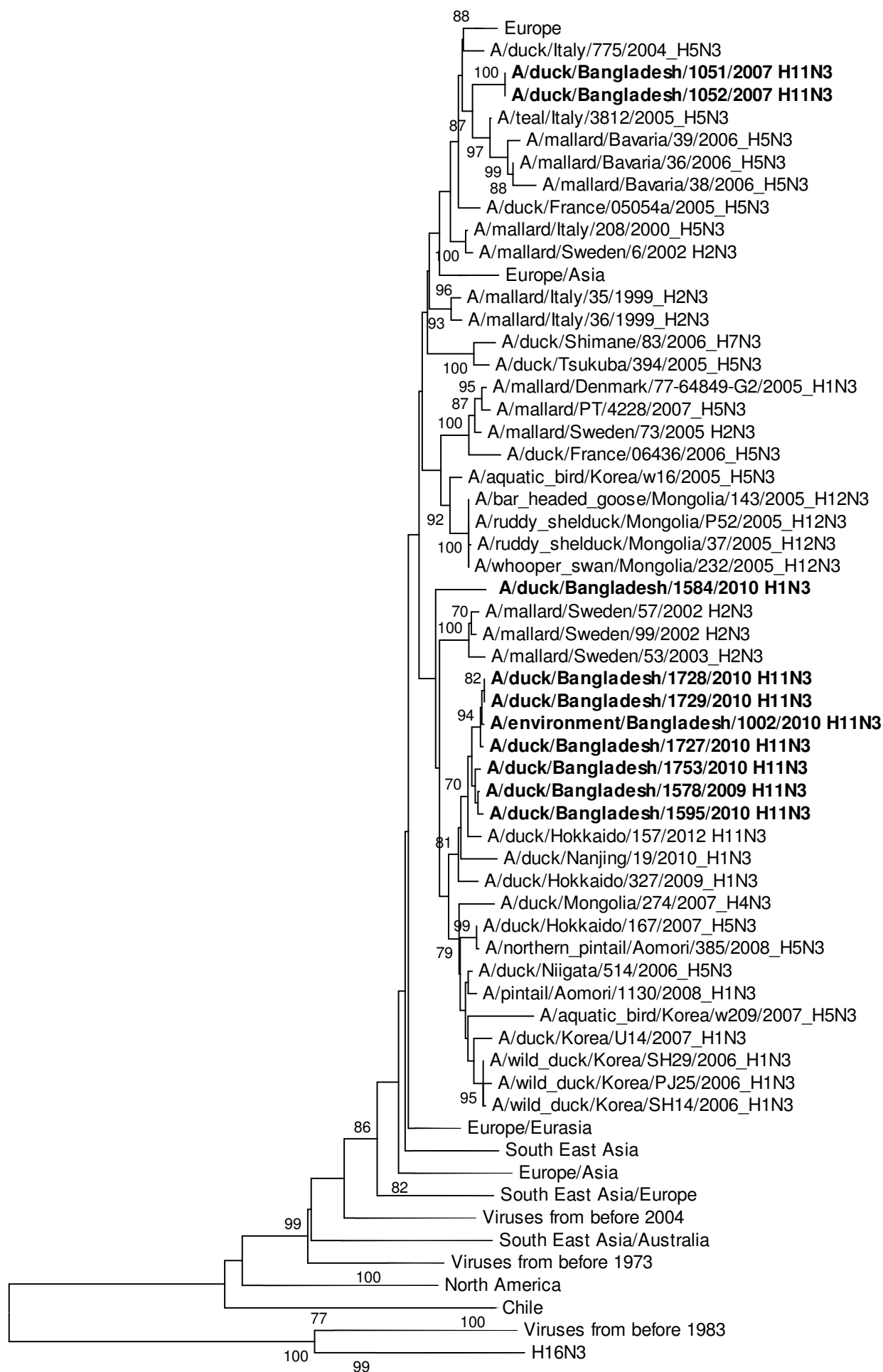

E

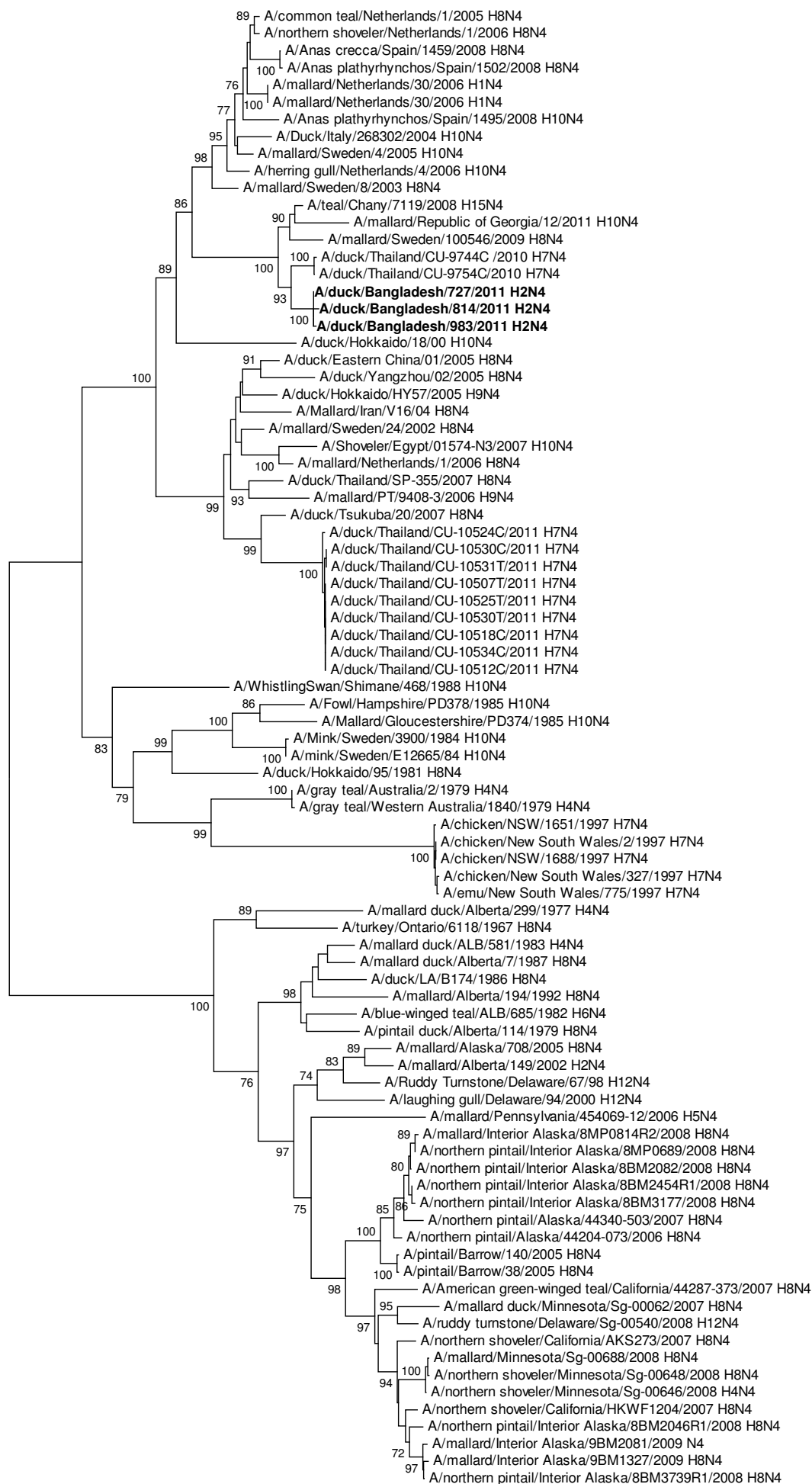

0.02

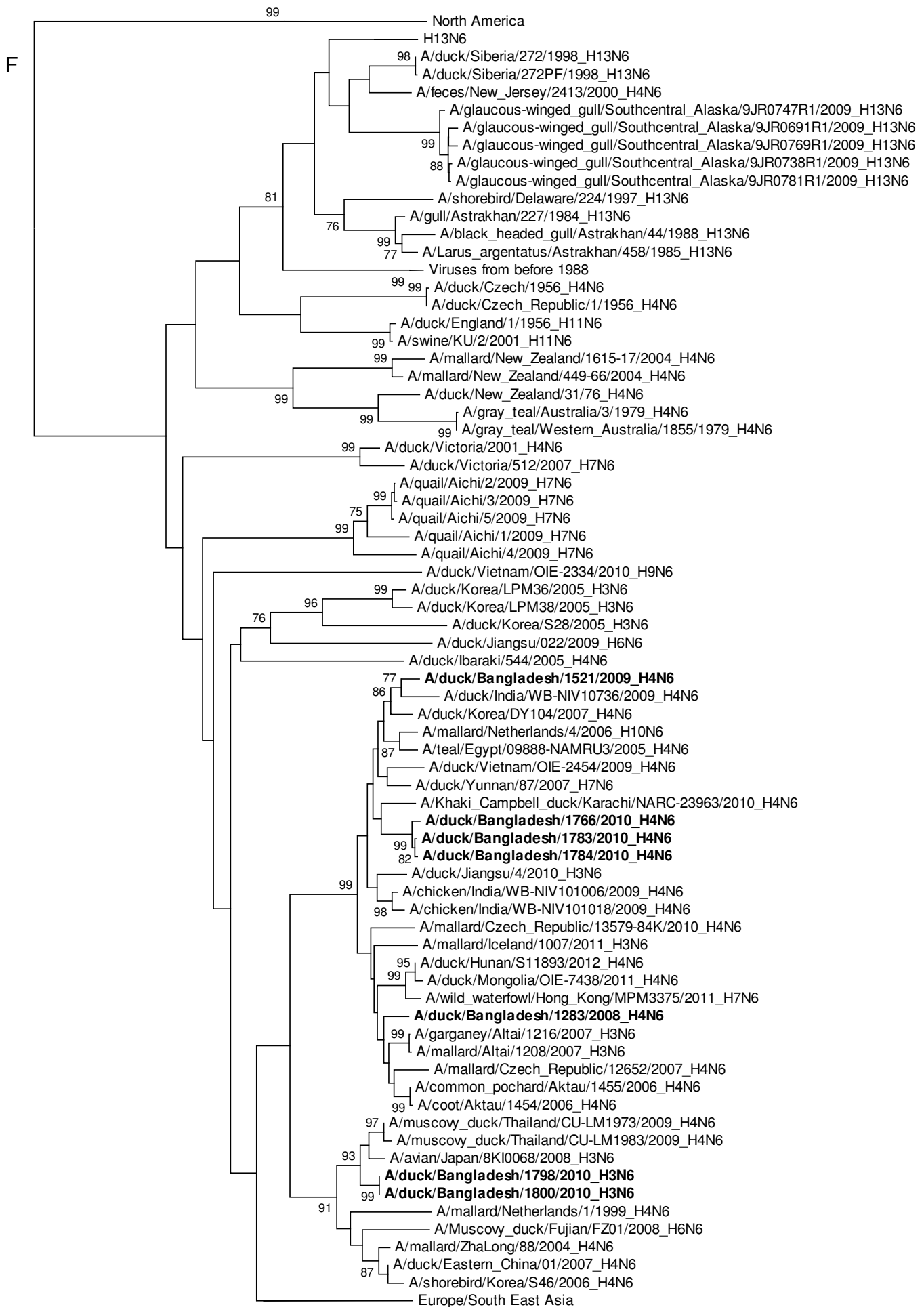

G

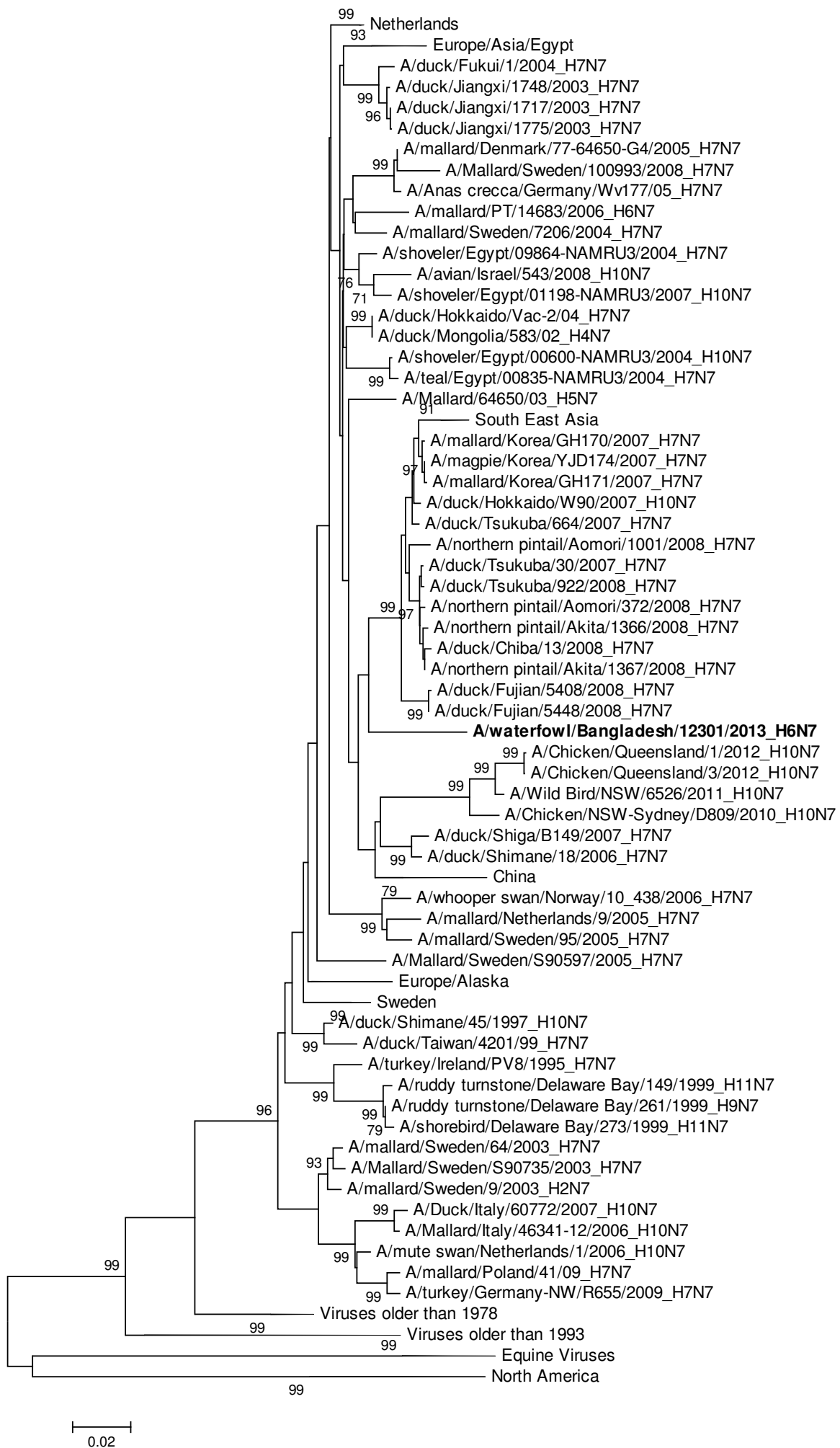



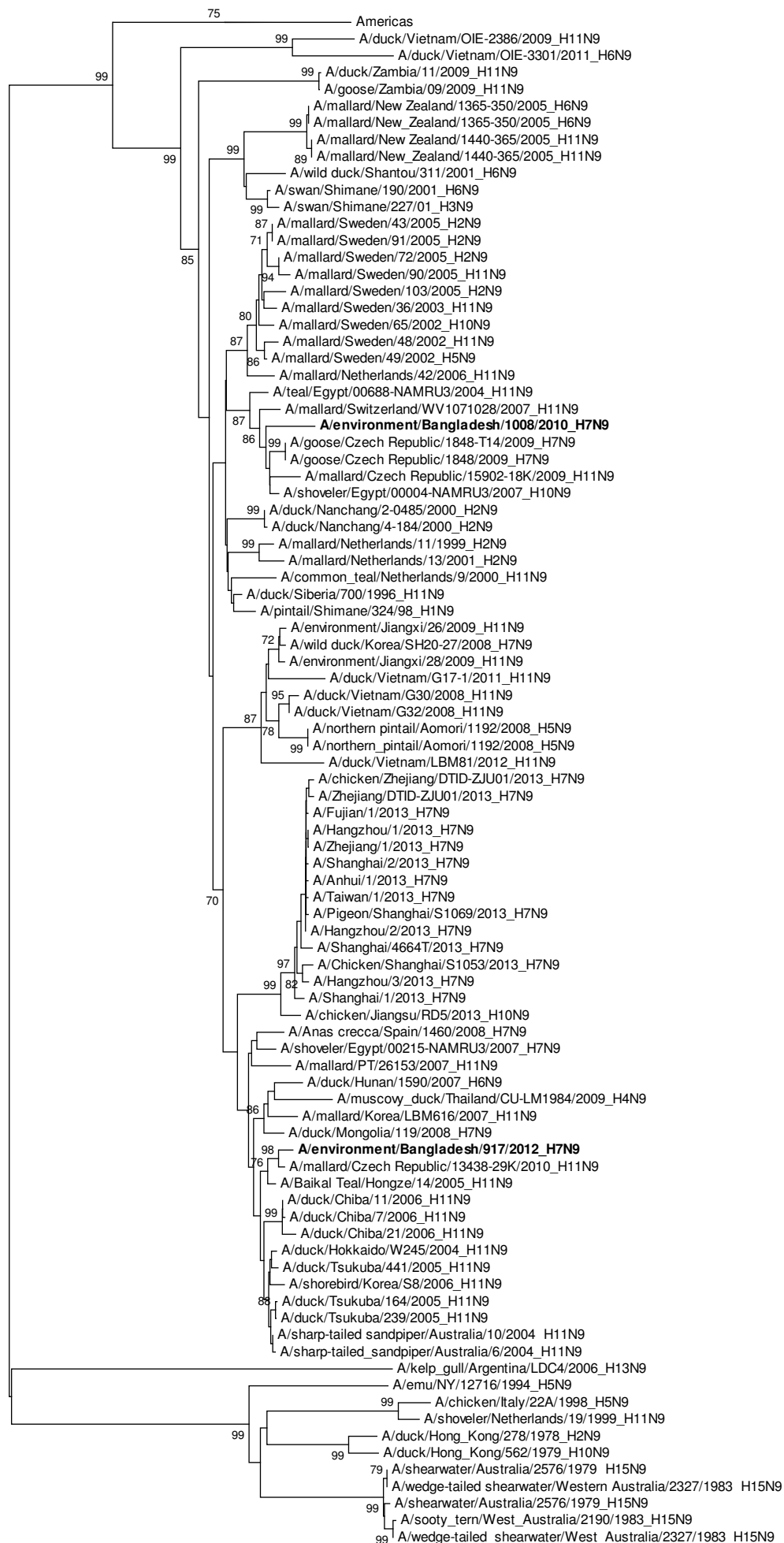

0.02

Supplement: S1 Fig — Phylogenies of the complete coding neuraminidase genes for subtypes N1 (A), N2 (B), N2 of H9N2 viruses (C), N3 (D), N4 (E), N6 (F), N7 (G), N8 (H) and N9 (I). The viruses identified in this study are shown in boldface. For clarity large branches were collapsed and labeled according to the geographic location or collection years of viruses in that branch. Bootstrap values ≥70 are shown on branches. (PDF) [file pone.0152131.s001.pdf]

# S2 Fig

A

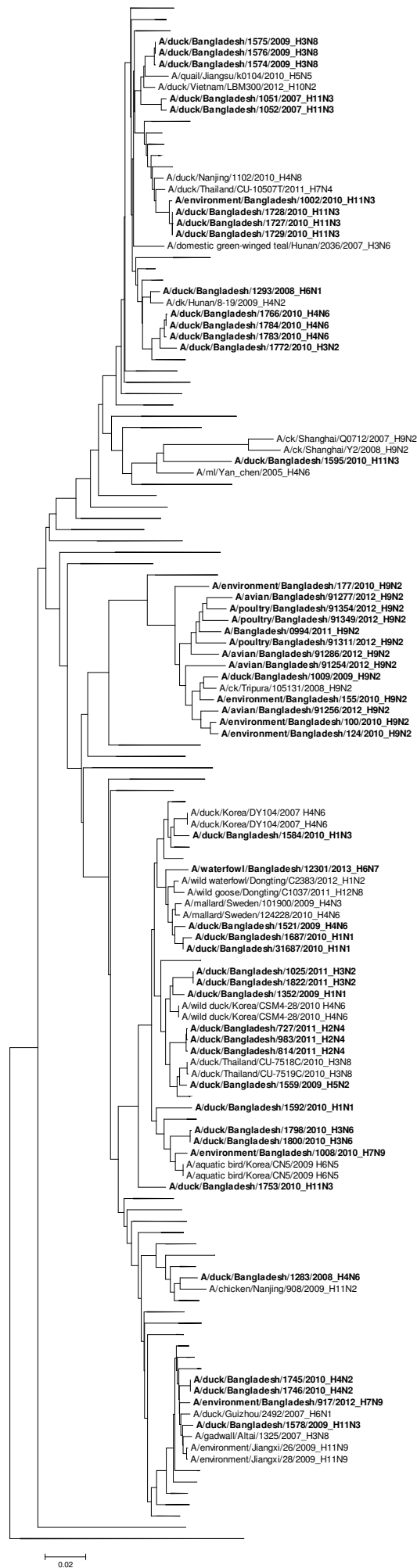

B

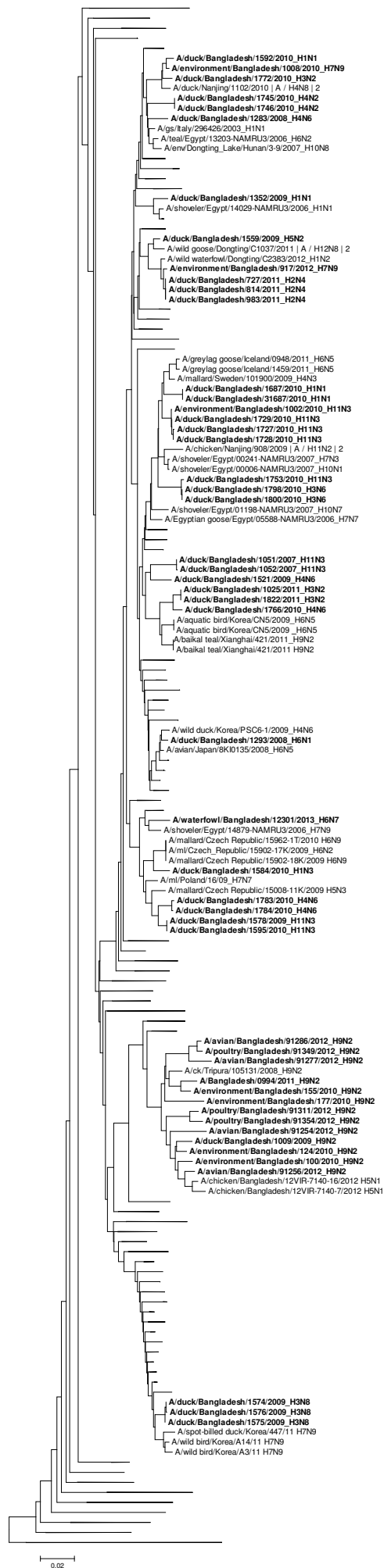

C

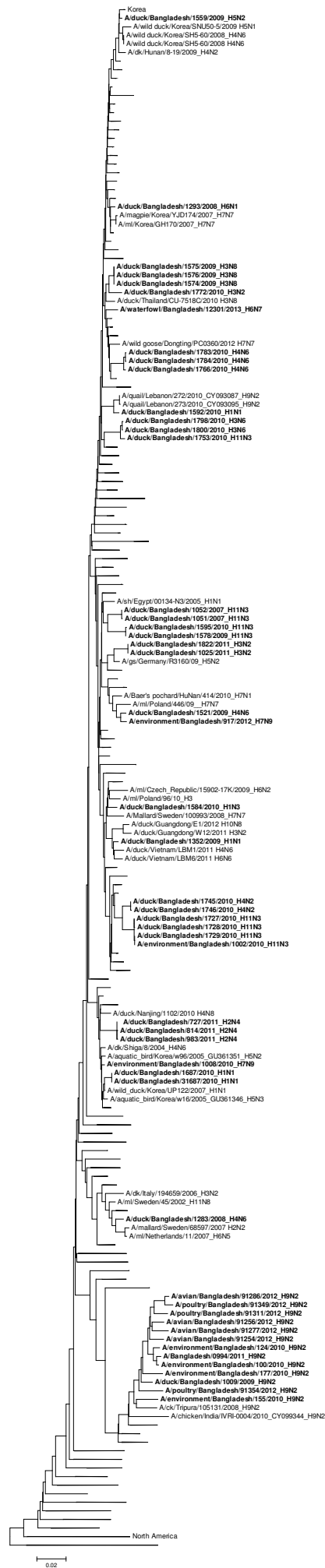

D

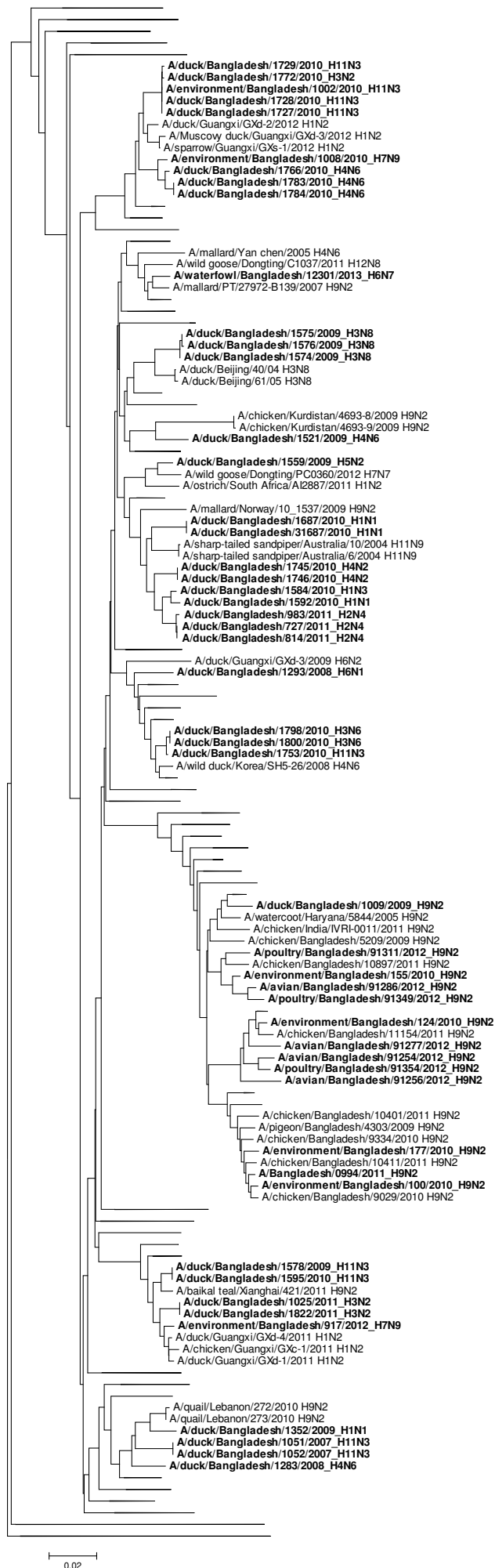

E

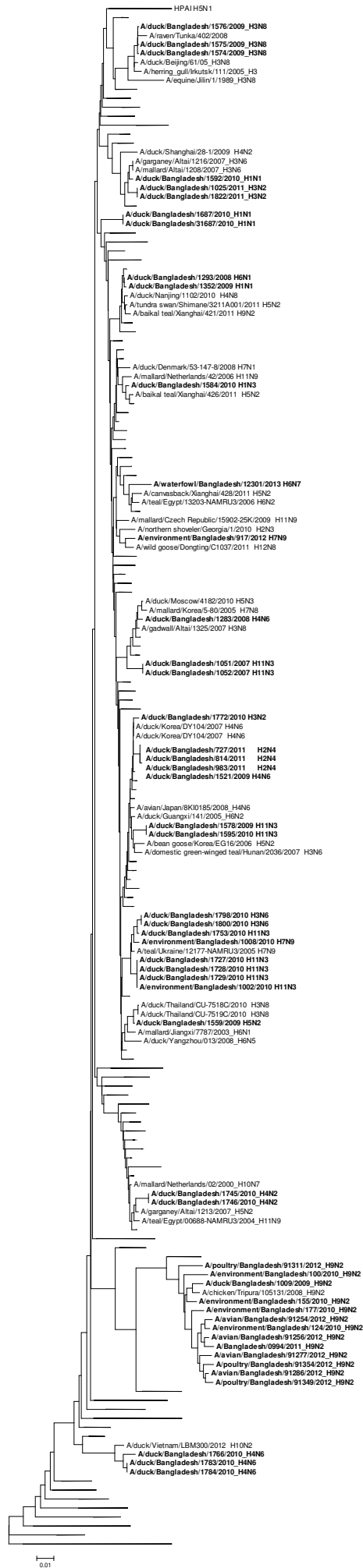

F

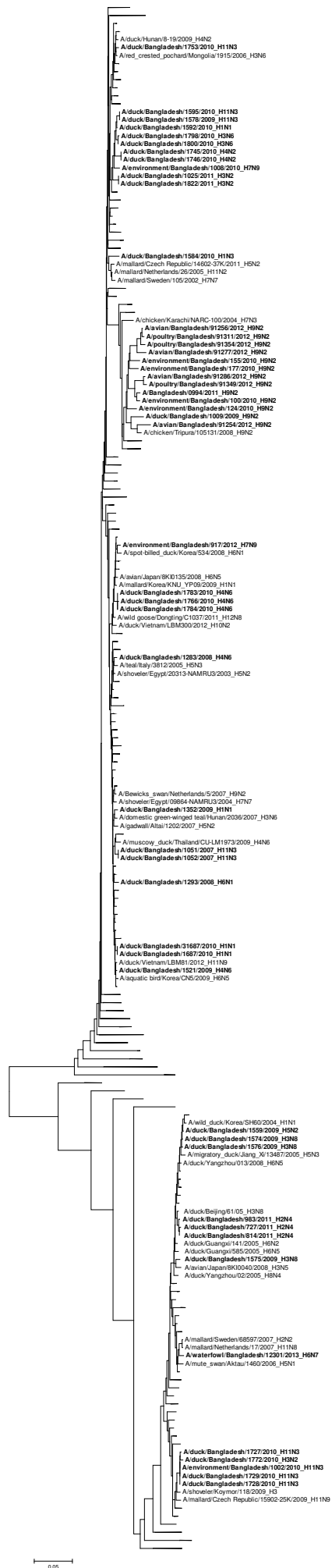

Supplement: S2 Fig — Phylogenies of complete coding internal genes (A) polymerase basic 2 (PB2), (B) PB1, (C) polymerase acid (PA), (D) nucleoprotein (NP), (E) matrix protein (M), and (F) nonstructural proteins (NS). The viruses identified in this study are shown in boldface. For readability, strain names were removed for viruses that did not cluster with Bangladesh strains and/or large branches were collapsed and labeled according to the geographic location of viruses in that branch. (PDF) [file pone.0152131.s002.pdf]
